# Beyond *In Vivo*, Pharmaceutical Molecule Production in Cell-Free Systems and the Use of Noncanonical Amino Acids Therein

**DOI:** 10.1021/acs.chemrev.4c00126

**Published:** 2025-01-22

**Authors:** Marco G. Casteleijn, Ulrike Abendroth, Anne Zemella, Ruben Walter, Rashmi Rashmi, Rainer Haag, Stefan Kubick

**Affiliations:** † VTT Technical Research Centre of Finland Ltd, 02150 Espoo, Finland; § Fraunhofer Institute for Cell Therapy and Immunology (IZI), Branch Bioanalytics and Bioprocesses (IZI-BB), Am Mühlenberg, 14476 Potsdam, Germany; ∥ Freie Universität Berlin, Institute of Chemistry and Biochemistry, 14195 Berlin, Germany; ⊥ Faculty of Health Sciences, Joint Faculty of the Brandenburg University of Technology Cottbus–Senftenberg, The Brandenburg Medical School Theodor Fontane and the University of Potsdam, 14469 Potsdam, Germany; # B4 PharmaTech GmbH, Altensteinstraße 40, 14195 Berlin, Germany

## Abstract

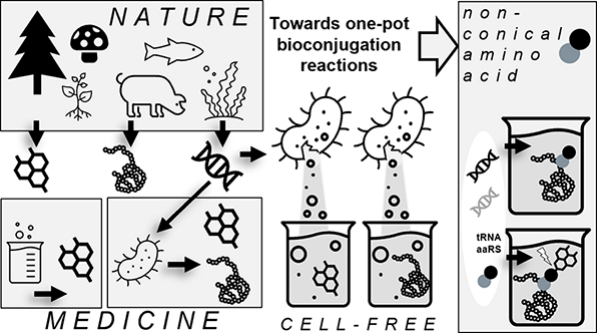

Throughout history, we have looked to nature to discover and copy pharmaceutical solutions to prevent and heal diseases. Due to the advances in metabolic engineering and the production of pharmaceutical proteins in different host cells, we have moved from mimicking nature to the delicate engineering of cells and proteins. We can now produce novel drug molecules, which are fusions of small chemical drugs and proteins. Currently we are at the brink of yet another step to venture beyond nature’s border with the use of unnatural amino acids and manufacturing without the use of living cells using cell-free systems. In this review, we summarize the progress and limitations of the last decades in the development of pharmaceutical protein development, production in cells, and cell-free systems. We also discuss possible future directions of the field.

## 1 Introduction

Pharmaceutical molecules, including proteins, are central to the treatment or prevention of diseases in humans, pets, and livestock. There are several important aspects underscoring their importance. In disease treatment, pharmaceutical molecules are designed to interact with specific biological targets in the body to either inhibit or enhance their functions. This can include small-molecule drugs, biologics (including proteins such as antibodies), and gene therapies. They are used to treat a wide range of diseases and conditions, from common illnesses like the flu to chronic conditions such as diabetes and autoimmune diseases.

Since the early attempts by Edward Jenner in 1796,^1^ vaccines, which often contain proteins or other biological molecules, are essential for preventing infectious diseases. They stimulate the immune system to produce antibodies or other immune responses, providing protection against future infections. In more recent years, precision medicine has been developed due to the advances in molecular biology and genomics.^2^ These personalized medicines are tailored to an individual’s genetic makeup, ensuring more effective and safer treatments with fewer side effects. Proteins and nucleic acid-based (DNA and RNA) drugs play critical roles in precision medicine approaches.

Ever since humans settled down and started living with their livestock, infectious diseases rose hand in hand with the growing population.^3^ As such, veterinary medicine has become important not only from an economic point of view but also from a public health point of view. One current example is bird flu, which negatively impacts the agricultural industry and raises health concerns about cross-contamination to our pets or humans.^4^ Pharmaceuticals are crucial in veterinary medicine for the treatment and prevention of diseases in pets and livestock.

Therefore, having access to essential pharmaceuticals is a critical aspect of global health. Ensuring the availability and affordability of these molecules is a key component of public health efforts worldwide.^5^ As such, advances in biotechnology and chemistry to study disease mechanisms, identify potential drug targets, and develop new therapeutic approaches are crucial to adapt rapidly to new pandemics or address global health threats, such as diabetes, sepsis, cancer, and cardiac diseases, to name a few. Proteins, such as enzymes and antibodies, are essential tools in laboratory experiments and diagnostics. An additional element is the speed and scalability of production for rapidly emerging health threats, especially on a global scale. Currently, access to essential medicines for the world’s poor has made little progress, except for a few medicines such as antiretrovirals.^5^

This review is organized into five sections. In section 2, we present the current state of the art regarding pharmaceuticals currently on the market and the nature of pharmaceutical molecules. In section 3, the use of cells and their lysates in cell-free protein synthesis (CFPS) to manufacture pharmaceuticals is reviewed. In section 4, we provide a perspective on noncanonical amino acids, specifically on their role in bioconjugations and cell-free protein synthesis systems. In section 5, we bring together the different methods, to finalize in the last section a general discussion and insights on where the field is going next.

## 2 Pharmaceutical Molecules

Pharmacy, as part of medicine, has its roots in centuries of experimenting with cool water, leaves, dirt, herbs, nuts, plants, or even mud during prehistoric times.^6^ These efforts were subsequently summarized in recent millennia, first in writings in Mesopotamia and Egypt, and later in early attempts at pharmacopeia by Pedanius Dioscorides (in “De materia medica”),^7^ Jiang Shinian a.k.a. Shennong (in “Shennong Bencaojing”) and others,^8^ Abu Rayhan Muhammad ibn Ahmad al-Biruni a.k.a. al-Biruni (in “Kitab al-Saydalah”),^9^ and Nicolaus Salernitanus or others (in “Antidotarium Nicolai”).^10^ However, it was not until scientists started extracting single molecules from such early pharmaceutical preparations that modern pharmaceutical science emerged.

Morphine, discovered and isolated by Friedrich Sertürner (1783–1855), is commonly accepted as the first medicinal alkaloid isolated from plants.^11^ Thereafter, many pharmaceutical compounds were isolated in the decades that followed,^11^ including extracts from animals, such as epinephrine.^12^ It was not until around 1831 that the first pharmaceutical compound was chemically produced: chloroform.^13^ Over 90% of the approximately 19,000 prescription drug products approved by the Food and Drug Administration (FDA) on the market^14^ are small molecular drugs,^15^ even though the current major blockbusters are mainly biopharmaceutical proteins.^16^

Pharmaceutical proteins followed a similar path.^17,18^ Active pharmaceutical proteins were discovered either from early potions or subsequently from plants or isolated from animals. The first pharmaceutical proteins were mixtures of polyclonal antibodies, described as serum therapy for the treatment of diphtheria.^19,20^ This was followed by the first administration of a purified protein on January 11, 1922 when insulin, isolated from ox-pancreas extract, was injected into Leonard Thompson, a 14-year-old diabetic.^21^ Insulin was also the first protein sequenced by Frederick Sanger in 1949.^22^ With the discovery of DNA and the advances made in molecular biology, it was also insulin that made it to the market in the 1980s as the first recombinant pharmaceutical protein produced *in vivo*; in this case in the microbe *Escherichia coli*(21) (*E. coli*).

The first pharmaceutical proteins on the market that combined the specificity of pharmaceutical proteins with the additional properties of a chemical moiety by linking them together were PEG-adenosine deaminase (for the treatment of acute immunodeficiency syndrome) and PEG- l-asparaginase (for the treatment of acute lymphoblastic leukemia).^23^ Poly(ethylene glycol) (PEG), a polymer, is used in these applications to mask pharmaceutically active proteins from the immune system. Another early example is stryene-*co*-maleic anhydride conjugate of the anticancer protein neocarzinstatin with the purpose of solubilizing the protein in the phase contrast agent Lipiodo.^23^

The development of small molecular drugs and proteins thus followed similar paths, from extracting pharmaceutical agents from plants and animals to manufacturing them using synthetic routes. The advantageous properties of small molecules and proteins were then combined to create engineered, highly specific pharmaceuticals.

### 2.1 *In Vivo* Pathway Engineered Production of Small Molecular Drugs

The focus of this review is on protein-based drugs; however, due to the potential converging nature of the production of small molecule drugs and pharmaceutical proteins by means of biotechnology, conjugation chemistry, and synthetic biology, we will briefly discuss the *in vivo* manufacturing of small molecules through metabolic pathway engineering. Traditionally, small molecule drugs are produced without the use of biotechnology.^24,25^ However, in the mid-1970s biological cells were quickly adapted to produce chemicals, fuels, proteins (including enzymes), and pharmaceuticals due to the work of Cohen and Bailey.^26,27^ Metabolic engineering was then defined in 1991 as “the improvement of cellular activities by manipulation of enzymatic, transport, and regulatory functions of the cell using recombinant DNA technology”.^27^

**Figure 1:**
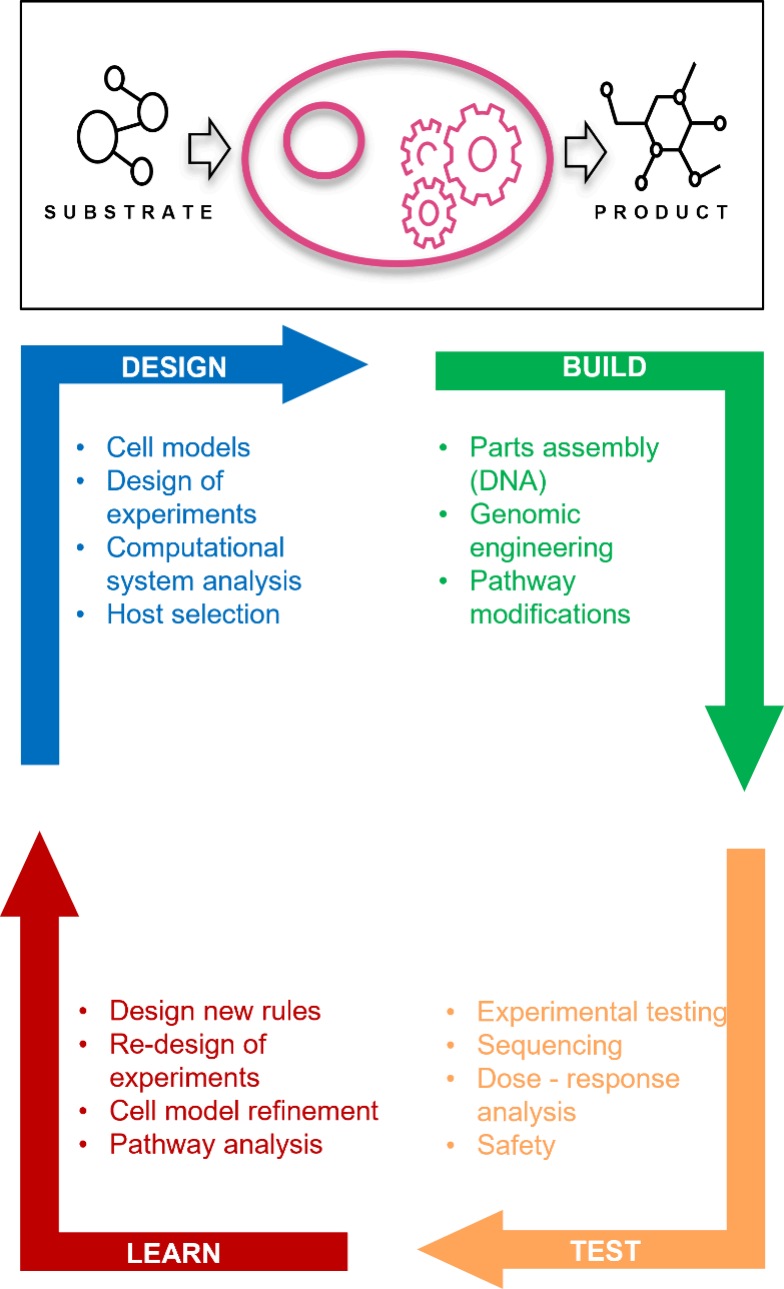
In order to convert a conventional cell into a cell that can be utilized for the production of compounds, including proteins, cells are (re)designed using the Design/Build/Test/Learn (DBTL)-cycle. Cells used for the manufacturing of compounds, including proteins have been referred to as cell factories.^29^ To produce small molecular drugs, enzymatic pathways are engineered or deleted in the cell, utilizing several rounds of the DBTL-cycle to optimize the production. Single enzymes, or a cascading pathway of enzymes, can be engineered to optimize the use of substrates to maximize the output of product. In the case of protein production, feedstocks containing carbon, nitrogen, and other essential elements needed for cell-growth/viability are considered the substrate which enters the cell, while the product is (secreted) protein.

Metabolic engineering has proven to be successful for the production of small molecule drugs.^28^ However, when large-scale production is targeted, a typical manufacturing process first selects which drug to produce. Then, the most suitable microbial host strain is selected based on its metabolic characteristics and capabilities to produce the drug, the ease of culturing the host strain, and the availability of genetic engineering tools. Ideally, the process is supported by computational simulations and high-throughput omics (protein, DNA, RNA, lipids, carbohydrates, and metabolites) analyses to map metabolic and cellular networks and predict metabolic phenotypes at the levels of transcripts, proteins, metabolites, and flux under various bioprocessing conditions. Metabolic engineering is performed by optimizing existing pathways, establishing new pathways, and, if necessary, adding regulatory circuits. Fermentation and downstream processing (DSP) of the engineered host strain are followed to produce the desired drug of interest. Further optimizations of the host strain follow the Design/Build/Test/Learn (or DBTL)-cycle (Figure 1) to maximize the output (i.e., product) with minimal input (substrate). This subprocess is often needed to increase yield, purity, and stability of the product. The final industrial robust production strains are then scaled up even further for commercial drug production.

Genetic and metabolic engineering achievements for the production of drugs and drug precursors are well summarized in recent reviews.^30,31^ In summary, the field has made significant strides over the last few decades in the production of nonprotein-based pharmaceutical compounds. In the 1980s, metabolic engineering was used to enhance the production of antibiotics in microbial hosts other than fungi. The first pharmaceutical molecule produced at an industrial scale was semisynthetic artemisinin, a potent antimalarial drug.^32^ Other important examples include the production of paclitaxel (Taxol)^30^ and artemisinic acid in yeast as a precursor to artemisinin^33^ in the 2000s, the production of opioid precursors and morphine in yeast and bacterial strains,^34,35^ and with the rise of personalized medicine,^2^ engineered precision medicine has the potential to be an effective healthcare approach.^36^

These achievements illustrate the evolving capabilities of metabolic engineering in the production of nonprotein-based pharmaceutical compounds.

### 2.2 Specific Nature of Pharmaceutical Proteins

Biologics (or biodrugs) are defined by the FDA and the European Medicines Agency (EMA) as “made from a living organism or its products and is used in the prevention, diagnosis, or treatment of cancer and other diseases. Biological drugs include antibodies, interleukins, and vaccines. Biologics can also be referred to as biologic agents or biological agents”. Vaccines, blood-products, DNA or RNA base therapeutics (e.g., siRNA, aptamers, mRNA), pharmaceutical proteins, gene therapy, stem cell therapy and tissue engineering, and extracellular vesicles all classify as biologics.

The estimated total sales volume of the pharmaceutical industry in 2021 was 1.42 trillion USD,^37^ of which 336.5 billion USD was attributed to pharmaceutical proteins.^16^ Due to the COVID pandemic, among the 15 major blockbusters in 2021, there were two mRNA vaccines (Pfizer’s Comirnaty and Moderna’s Spikevax COVID-19 vaccines) on places 1 and 3, with two small molecules on places 5 and 10 (Bristol Myers Squibb and Pfizer’s Eliquis (apixaban) and Gilead Sciences’ Biktarvy (bictegravir, emtricitabine, and tenofovir alafenamide), respectively.^38^ The remainder being pharmaceutical proteins underscores the importance of this class of therapeutics. Small molecule drugs have several disadvantages compared to those of pharmaceutical proteins. Pharmaceutical proteins can perform complex functions and are highly specific, which is not possible for small molecule drugs. Due to the higher specificity, pharmaceutical proteins are less likely to exhibit drug toxicity. In addition, they are less likely to elicit an immune response since they are either recombinant versions of proteins naturally produced by the human body or engineered to be human-like (humanized proteins; Table 1). Pharmaceutical proteins are also suitable alternatives to gene therapy when such options are not (yet) available. The other side of the coin is that pharmaceutical proteins are rarely available as oral drugs, the production costs are higher than small molecule drugs, and inefficient penetration into tissues to reach the target site can be challenging if the pharmaceutical protein is too large.^39^

**Table 1:** Examples of the Pharmaceutical Proteins

Generation	Brand name (Generic name)	Modifications	Therapeutic category (Indication)	Manufacturer
first	Humulin (Insulin)	-	Diabetes (Diabetes)	Elli Lilly
	Hepatrope (Somatropin^a^)	-	Hormones (Growth failure)	Elli Lilly
	Intron A (Interferon α-2b)	-	Anti Infective (Viral Infections)	Schering-Plough
	Procrit/Eprex (Epoetin α^b^)	-	Blood modifier (Anemia)	Johnson and Johnson
	Kogenat (Factor VIII)	-	Blood modifier (Hemophilia)	Bayer
second	Humalog/Liprolog (Insulin Lispro)	Protein engineered (K/P swap in B chain of the insulin molecule)	Diabetes (Diabetes)	Elli Lilly
	Pegasys (Interferon α-2b)	PEGylated	Interferon (Hepatitis C)	Roche
	Refacto (Factor VIII)	B-domain-deleted rh factor VIII	Blood modifier (Hemophilia)	Wyeth
	Amevive (Alefacept)	Dimeric fusion protein (extracellular CD2-binding portion of human LFA-3 linked to the Fc region of human IgG1)	Inflammation/Bone (Plaque psoriasis)	Biogen Idec^c^
	Ontak (denileukin diftitox)	Recombinant r IL-2–diphtheria toxin fusion protein	Cancer (Cancer)	Ligand Pharmaceuticals^c^
third	Gazyva (US) Gazyvaro (EU) (Obinutuzumab)	Humanized, glycoengineered mAb specific for B cell antigen CD20	Chronic lymphocytic leukemia (Cancer)	Roche/Genentech
	Synagic (Palivizumab)	humanized mAb	Prophylaxis of lower respiratory tract disease (Viral infection)	AstraZeneca
	Emicizumab (EU), Emicizumab-kxwh (US) (Hemlibra)	Humanized, bispecific IgG4 capable of binding factors IXa and X	Blood modifier (Hemophilia)	Roche Registration (UK) Roche/Genentech (USA)
	Belantamab mafodotin (EU), Belantamab mafodotin-blmf (US) Blenrep	ADC comprising monomethyl auristatin F conjugated to an afucosylated humanized IgG1κ targeting B cell maturation antigen	Multiple melanoma (Cancer)	GlaxoSmithKline
	Zynlonta (loncastuximab tesirine-lpyl) CD19-directed humanized IgG1κ produced in a CHO cell line, conjugated to SG3199 (alkylating agent).	CD19-directed humanized IgG1κ conjugated to SG3199 (alkylating agent)	Lymphoma (Cancer)	ADC Therapeutics

a Somatropin was also produced by Pfizer as Genatropin and by Serono as Saizen.

b Epoetic α was also produced by Amgen as Epogen and by Roche as NeoRecormon.

c Withdrawn or discontinued. mAB = monoclonal antibody.

When considering pharmaceutical proteins, we must understand the complex nature of their structure, their production within cells, and their proper folding into biologically active molecules.^40^ Another challenging aspect of recombinant protein production is that production hosts are often not of the same genetic origin as the target protein, e.g., the production of human insulin in yeast cells. This requires extensive host engineering and optimization of the production process parameters. Some proteins are additionally modified, e.g., glycosylation, often through expression hosts engineered to mimic human post-translation modification (PTMs). Such PTMs may affect protein stability, solubility, or efficacy during manufacturing, storage, and during or after administration.

Protein-based drugs can be classified in different generations of biotechnology drugs: natural biopharmaceuticals, e.g., proteins obtained by extractive processes and natural proteins obtained by recombinant DNA technology (1st generation; recombinant proteins), modified recombinant natural proteins e.g., point mutation(s), hyperglycosylation, PEGylation (2nd generation; displaying the same biological activity, similar or enhanced clinical efficacy, efficacy/safety ratio and PK/PD profile), and highly modified recombinant proteins, e.g., multiple mutations, chimers, fusion proteins (3rd generation; new molecules with different activity and clinical application than the deriving natural proteins). In Table 1, some examples of pharmaceutical proteins are listed. Additional examples can be found in a recent book chapter^41^ and the full list of biological therapeutics with market approval is well summarized by Walsh (2022).^16^

Another important parameter to consider is the metabolic clearance of pharmaceutical proteins. Unlike small molecules, which are broadly metabolized by cytochrome P450 enzymes, mainly in the liver, proteins are digested by proteases that are found throughout the body, in blood, various organs, tissue, lymphatic fluid, interstitial fluid, and intracellularly. Typically, smaller proteins or digested subunits (< ∼50 kDa) are eliminated primarily via the kidneys, with high levels of renal filtration and (additional) degradation after proximal tubule reabsorption. For larger proteins, such as antibodies, both receptor-mediated (or active) and fluid-phase endocytosis (or passive) mechanisms are the main elimination mechanisms, transporting them from the vascular endothelium to the underlying tissue and intracellular degradation.^42^

Pharmacogenetics can play an important role in identifying responders and nonresponders to medications, avoiding adverse events, and optimizing drug doses. Pharmacogenetic information and changes in drug labeling are expected to accelerate protein engineering for pharmaceutical proteins targeting different populations, personalized dosing regimens, and companion diagnostics.^43^

When considering the progression and constant improvement of protein engineering tools, it is reasonable to expect more complex pharmaceutical proteins in the future. This would also include proteins with sequences not found in nature.^43^ Protein engineering of pharmaceutical proteins has focused on improving the stability and half-life of therapeutics after administration to the patient. In addition, masking pharmaceutical proteins from the native immune system to improve their half-life and avoid adverse effects is another important area. As such, careful design is warranted for immunogenicity risk assessment and mitigation, especially with the advancements in computational tools and off-the-shelf platform technologies combined with novel protein structures including conjugated molecules. Another critical note regarding the expansion of the design space and increasing high-throughput methods, is the challenge of detecting anomalies in data sets and the growing number of internal parameters due to an increase in AI models.^44^ As such, a tighter collaboration is needed between computational scientists and protein drug developers. In addition, with the expansion of novel drugs a re-examination of risk mitigation strategies for biologic treatments, especially via postmarket surveillance is necessary.^45^

### 2.3 Modifications of Pharmaceutical Proteins

Classical protein engineering comprises making changes to the DNA sequence to change the protein structure to alter the stability and binding properties, or, in the case of enzymes, catalytic properties. Structural predictions have been used to guide rational design, while randomized methods followed by screening have proven to be very powerful in the past.^46,47^ However, nowadays protein engineering can span a wide range of modifications, such as conjugations (PEG, POX, PASylation, fatty acids, gene manipulations, (pre- or post-translational^48−50^) protein fusions (e.g., F_c_-fusion), amidation, or disulfide bond shuffling. As such, molecular modeling^51,52^ in combination with the state-of-the-art protein folding predictions, e.g., Alphafold^53,54^ and RoseTTAFold,^55^ could be a powerful new avenue to be used in *grafting* approaches. Three grafting routes are utilized to prepare biomolecule-polymer conjugates in a controlled manner: *grafting-to* in which a polymer is first synthesized, purified, and then attached, *grafting-from* where a small, reactive molecule is the initiation site to grow the polymer from the surface of the protein, and *grafting-through* where monomers tailored with a precise payload are polymerized.^56^ For pre-expression protein engineering, we refer to other excellent reviews in the field.^57−59^

Macromolecular drugs synthesized by attaching a therapeutic molecule to either a lipid or a polymeric carrier molecule using covalent chemical linkers are called bioconjugated therapeutics and can be seen as post-translational protein engineering. Such macromolecules are composed of three basic building blocks: a carrier molecule (polymer, lipid, peptide, mRNA, or protein), a therapeutic agent (small molecule chemicals or macromolecular drugs), and chemical linkers. Bioconjugate therapeutics are considered macromolecular prodrugs (a compound with little or no pharmacological activity that converts into a pharmacologically active drug compound in the body) since the therapeutic agents are covalently conjugated to carrier molecules. Table 2 lists all pharmaceutical proteins with bioconjugated moieties approved by the FDA and/or EMA up to 2022.

**Table 2:** Bioconjugated Pharmaceutical Proteins Previously Approved by the FDA/EMA^a^

Brand name^16^ (Generic name)	Conjugating method	Indication^16^	Manufacturer^16^
Mylotarg (gemtuzumab ozogamicin), ADC targeting the CD33 surface antigen, consisting of a humanized IgG4 chemically conjugated to N-acetyl-γ-calicheamicin.	Anti-CD33 antibody carbohydrate is conjugated with NAc-gamma calicheamicin DMH made by oxidizing the naturally occurring carbohydrate residues and reacting the resultant aldehydes with the calicheamicin hydrazide derivative^70^	Acute myeloid leukemia	Pfizer (Belgium) Pfizer/Wyeth (USA)
Adcetris (brentuximab vedotin), chimeric mAb conjugate specific for human CD30 (expressed on the surface of lymphoma cells).	Monoclonal antibody linked with maleimide attachment groups, cathepsin-cleavable linkers (valine-citrulline), and para-aminobenzylcarbamate spacers to three to five units of the antimitotic agent monomethyl auristatin E (MMAE)^71^	Lymphoma	Takeda Pharma (Denmark) Seattle Genetics (USA)
Kadcyla (trastuzumab emtansine), humanized mAb specific for HER2 antigen conjugated to the small-molecule cytotoxin DM1.	Thiol-containing maytansinoids, which have methyl groups adjacent to their sulfhydryl group, were linked tothe antibody trastuzumab with the SSNPP linker^72^	Breast cancer	Roche (Switzerland)
Ristempa (pegfilgrastim), covalent conjugate of rh G-CSF conjugated to 20-kDa PEG.	N-terminus methionine of filgrastim^73^	Neutropenia	Amgen (Netherlands)
Oncaspar (pegaspargase), r asparaginase and conjugated to monomethoxypropylene glycol.	PEGylation with a succinimidyl carbonate linker reacting with amine group of lysines and N-terminal amine^74^	Lymphoblastic leukemia, lymphoma	Les Laboratoires Servier (France)
Revcovi (elapegademase-lvlr), PEG-conjugated r bovine adenosine^75^ deaminase.	PEGylation with a succinimidyl carbonate linker reacting with amine group of lysines and N-terminal amine^76^	Adenosine deaminase severe combined immune deficiency (ADA-SCID)	Leadiant Biosciences (USA)
Polivy (polatuzumab vedotin), ADC comprising a humanized IgG1 targeting a component of the B cell receptor (CD79b) conjugated to monomethyl auristatin E (MMAE).	Maleimide addition to free (engineered) thiol groups (under reduced conditions) with a maleimidocaproylvaline-citrulline-*p*-aminobenzoyloxycarbonyl linker bound to monomethyl auristatin E^77^	Diffuse large B cell lymphoma	Roche Germany) Genentech (USA)
Givlaari (givosiran), chemically synthesized, chemically modified ds siRNA conjugated to a triantennary GalNAc ligand to facilitate hepatic delivery. Silences aminolevulinate hepatic synthase 1 (ALAS1) mRNA.	*trans*-4-hydroxyprolinol (*t*HP) moiety enabled site-specific conjugation at any position of an ON during solid-phase synthesis of siRNA^78^	Acute hepatic porphyria	Alnylam Netherlands (Netherlands) Alnylam (USA)
Besremi (ropeginterferon alfa-2b (EU), ropeginterferon alfa-2b-njft (US); rh-interferon alfa-2b with an additional N-terminal proline conjugated to a 40-kDa two-arm PEG moiety.	PEG aldehyde forms a tertiary amine linkage between PEG and pro-IFN alfa-2b with N-terminal proline^79^	Polycythemia vera	AOP Orphan Pharmaceuticals, (Austria) PharmaEssentia (USA)
Padcev (enfortumab vedotin (EU), enfortumab vedotin-ejfv (US)), antibody–drug conjugate (ADC) targeting nectin-4 (an adhesion protein highly expressed in urothelial cancer). Fully human IG1κ conjugated to monomethyl auristatin E (MMAE).	Maleimide addition to eight free thiol groups (under reduced conditions) with a peptide linker^80^	Urothelial cancer	Astellas Pharma Europe (Netherlands) Astellas Pharma US (USA)
Enhertu (trastuzumab deruxtecan), ADC comprising humanized anti-HER2 IgG1κ (trastuzumab sequence), conjugated to a topoisomerase I inhibitor derivative of exatecan.	MTGase mediated linker conjugation with dual click-chemistry drug conjugation (azide and me-tetrazine)^81^	Metastatic breast cancer	Daiichi Sankyo Europe (Germany) Daiichi Sankyo (USA)
Trodelvy (sacituzumab govitecan (EU), sacituzumab govitecan-hziy (US)), ADC comprising an anti-Trop-2 humanized IgG1κ conjugated to camptothecin-derived topoisomerase I inhibitor SN-38.	Maleimide addition to free thiol groups (under reduced conditions) with a short polyethylene glycol spacer containing an acetylene-azide facilitating click cycloaddition of SN38.^82^	Breast cancer (triple-negative)	Gilead Sciences (Ireland) Immunomedics (USA)
Blenrep (belantamab mafodotin (EU), belantamab mafodotin-blmf (US)), ADC comprising monomethyl auristatin F conjugated to an afucosylated humanized IgG1κ targeting B cell maturation antigen (BCMA).	Maleimide addition to the antibody of a protease resistant maleimido caproyl linker to a microtubule disrupting agent, mono methyl auristatin F (MMAF)^83^	Multiple myeloma	GlaxoSmithKline (Dublin) GlaxoSmithKline (USA)
Tivdak (tisotumab vedotin-tftv), tissue factor (TF)-directed ADC comprising a human anti-TF IgG1κ antibody conjugated to monomethyl auristatin E (MMAE), produced in a CHO cell line.	Maleimide addition to free thiol groups (under reduced conditions) with a peptide linker^84^	Cervical cancer	Seagen (USA)
Zynlonta (loncastuximab tesirine-lpyl), CD19-directed humanized IgG1κ conjugated to SG3199, an alkylating agent.	Maleimide addition to free thiol groups (under reduced conditions) with a dPEG8/Val-Ala peptide/PABA-linker^85^ conjugated to SG3199.	Lymphoma	ADC Therapeutics (USA)
Nexviazyme (avalglucosidase alfa-ngpt), rh α-glucosidase conjugated with multiple synthetic bis-mannose-6-phosphate (bis-M6P)-tetra-mannose glycans.	Recombinant hGAA was oxidized with sodium metaperiodate and to the hydrazine-derivatized M6P-containing oligosaccharides and phosphopentamannose^86^	Late-onset Pompe disease	Genzyme (USA)

a Medicine are in descending order of approval either by the FDA or EMA, based on Walsh, 2022.^16^

The set of chemical and enzymatic techniques utilized to attach moieties to proteins is extensive,^60^ therefore, selecting a correct strategy is very important.^61,62^ Bioconjugated pharmaceutical proteins are utilized to stabilize labile drugs from chemical degradation, to protect proteins from proteolytic degradation, to reduce immunogenicity, to decrease antibody recognition, to increase body residence time (i.e., increase half-life, for example in blood), to modify organ disposition, to facilitate drug penetration by endocytosis, to create new possibilities of drug targeting, and to deliver a drug (including the codelivery of a drug and mRNA).^63^

In cases where the protein cannot or does not have to be engineered to facilitate bioconjugation, the most straightforward and easy to perform techniques target natural amino acids.^64^ The most common targets are lysine, cysteine, and tyrosine. However, other natural amino acids have been reported.^65^ Side chains, and even terminal amine, provide accessible and reactive nucleophiles; therefore, these chemical groups are mostly used for nonspecific covalent bioconjugation strategies. However, the selection of natural amino acids is limited and selectivity and precision can be bottlenecks.

Lysine and amine strategies are popular since lysines are present in most proteins. Its primary amine is highly nucleophilic and very reactive toward electrophilic reagents, requiring activation. Several reagents are available, e.g., N-hydroxysuccinimides esters (NHS), sulfonyl chlorides, iso(thio)cynates, squaric acids, and vinyl sulfones. Of these, NHS esters used to form stable peptide bonds are the most common, also due to the commercial availability of these reagents.^65^ However, nonselectivity and pH dependency are drawbacks of this common method. Other reagents and their advantages and disadvantages are discussed in detail.^64,65^

Cysteine/thiol strategies have gained more traction in recent years, especially for functionalizing antibodies. Below a pH of 9.0 the cysteine’s thiol group is a stronger nucleophile compared to the primary amine of lysine.^65^ Overall, cysteine is less abundant than lysine, which can enhance the bioconjugation specificity but limit the payload. Cysteine can form disulfide bonds easily or can be alkylated with use of electrophiles, e.g., α-halocarbonyls and Michael acceptors, for example maleimides of vinyl sulfones. Here, the most popular reaction used for pharmaceutical proteins is the use of maleimides to form stoichiometric bioconjugates. A drawback is the need to use reducing agents prior to the conjugation reaction, which may affect the stability of the target protein.

Tyrosine possesses a phenolic hydroxyl group that can be targeted via a three component Mannich reaction with aldehyde and aniline reagents, diazonium salts for diazo arylation, or metal ion-catalyzed alkylation methods, such as palladium or nickel.^65^ The drawback in targeting tyrosine is that within the protein, they are often far less accessible than lysine or cysteine.

In addition, the bioconjugation of the carboxylic side chains of glutamic and aspartic acids is fairly common, since the carboxyl moieties are often present on the protein surface. These side chains can be activated with e.g., N,N-dicyclohexyl carbodiimine or 1-ethyl-3-(3-dimethylaminopropyl)carbodiimide (EDC) and reacted with amines to form a peptide bond. Other rare examples, such as histidine, methionine, and tryptophan have been reported.^65^

The development of antibody-drug conjugates (ADCs) has progressed with great strides over the past two decades. To date 15 ADCs have been approved by the FDA, the EMA, and other international governmental agencies.^66^ Additionally, hundreds of ADCs are being evaluated in preclinical and clinical phases.

ADCs also underwent several iterations, similar to other protein drugs. The first-generation ADCs had several disadvantages. Side effects were caused by immunogenicity of the mouse-derived and chimeric antibody itself, unstable linkers resulting in uncontrolled release of payloads, statistically coupled payloads resulting in different drug–antibody ratios (DAR) and low target specificity. For the second generation of ADCs, humanized mAbs were introduced to reduce the immunological response, and noncleavable, stable linkers were implemented to increase the stability of the protein-drug conjugates in blood. Moreover, more potent cytotoxic payloads were developed. Nevertheless, the heterogeneous DAR, off-target toxicity, and rapid clearance of the ADC were not resolved. With the third generation of ADCs, most of the disadvantages have been addressed. By use of fully human mAbs, immunogenicity is avoided. Furthermore, linker stability, payload toxicity, and conjugation strategies were improved. In particular, the shift from statistical to site-specific conjugation resulted in a more homogeneous DAR.

Nowadays, a variety of different technologies exist to produce antibodies with defined conjugation sites, such as cysteines,^67^ sugar modifications,^68^ and enzyme-based conjugation, such as transglutaminase for the introduction of small tags.^69^

## 3 Protein Synthesis Methodologies

The use of molecular biology techniques, and their constant advances during the last 60 years, have made recombinant protein expression the mainstream methodology for pharmaceutical protein production.^87−89^ The major reasons for the use of recombinant technologies to produce proteins are low availability of the native protein by means of extraction from natural sources, reproducibility of protein manufacturing in relation to its quality, immune responses to animal proteins after administration to patients,^90^ and infections of livestock used for the production of vaccines, and subsequent economic loss.^91^

Proteins can be produced in a variety of host organisms other than their own, such as bacteria, yeasts, molds, insects, protozoa, mammals, plants, and transgenic plants and animals. The gene of interest is inserted into the host organism, either as a plasmid (bacterial or yeast systems) or via genomic integration.^92^ A great deal of effort to enhance integration efficiencies and optimizing alternative integration mechanisms in recent years has diversified the selection of the production host.^92^ This is important, since choosing the correct expression system is mostly protein-dependent, and factors such as protein quality, functionality, production speed, and yield (titers) are the relevant parameters.^88,90,93^ Alternatively, protein production pathways can be isolated from cells and utilized in cell-free protein synthesis methods, sometimes referred to as ‘*In vitro* Transcription and translation (IVTT)’. In addition, chemical synthesis can produce proteins, however due to limitations in the size of the protein that can be produced and the costs of large-scale manufacturing, this technique has yet to be implemented for the production of pharmaceutical proteins.^94^

### 3.1 *In Vivo* Production of Pharmaceutical Proteins

**Figure 2:**
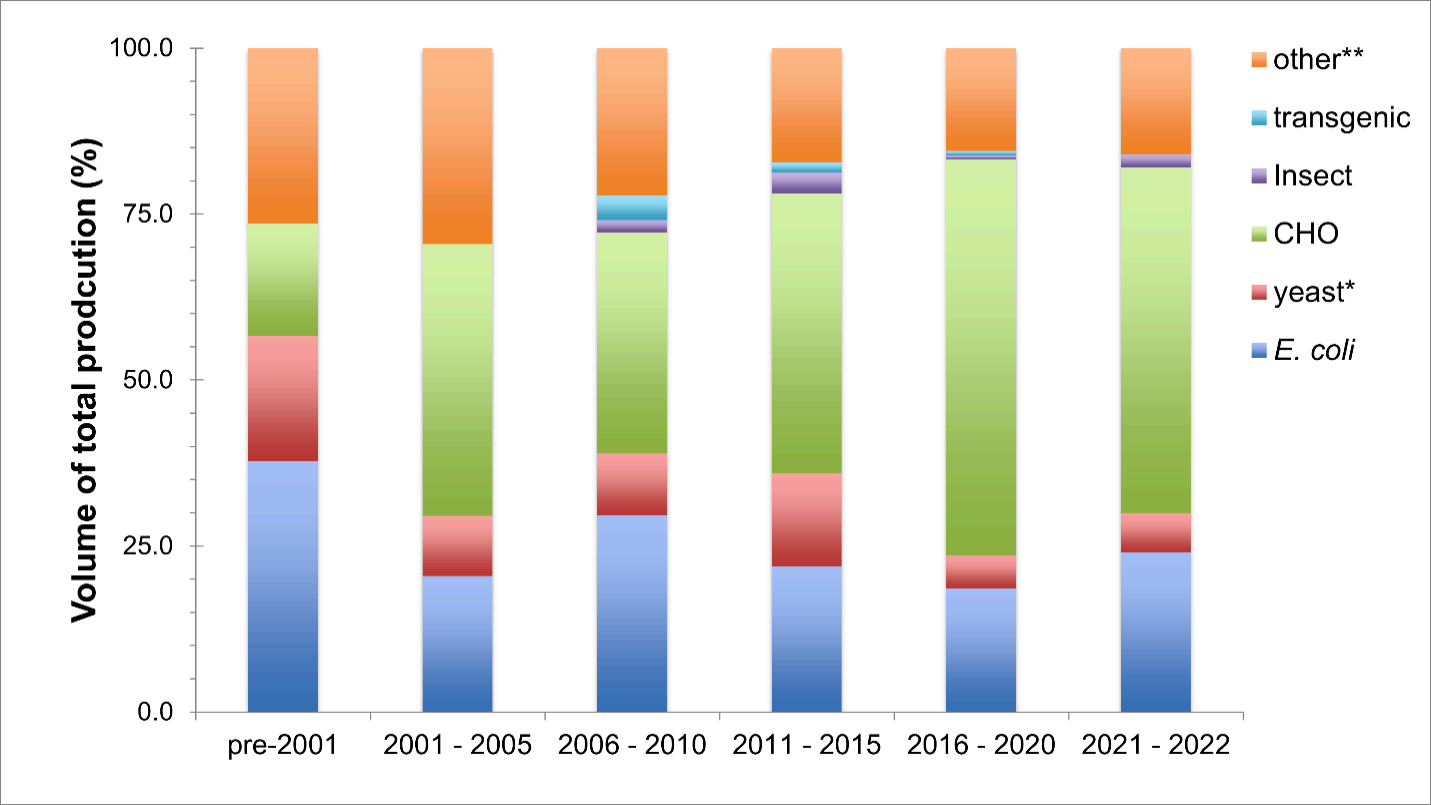
Distribution of expression systems used for the production of 497 market approved pharmaceutical proteins up to 2022. The amount per period is pre-2001, 106 proteins; 2001–2006, 44 proteins; 2006–2010, 54 proteins; 2011–2015, 64 proteins, 2016–2020, 161 proteins, and 2021–2022, 50 proteins. For the classification, the first approval date is taken (USA or EU), new approvals which were a combination of already approved were not included, gene therapy and nucleic acid biopharmaceuticals (other than mRNA vaccine using cell or cell-free systems for production) and engineered cell-based products were not included. *) Including *Saccharomyces cerevisiae*, *Komagataella pastoris* (*P. pastoris*), *Hansenula polymorpha*; **) including baby hamster kidney cells (BKH), murine cells, Sp2/0 cells, *V. cholera*, hybridoma cells, cell-free systems for mRNA vaccines, however the percentage represents mainly mammalian cells. Other abbreviations: Chinese Hamster Ovary Cells (CHO), *Escherichia coli* (*E. coli*), and transgenic animals include chickens (product in the eggs), rabbits (product in milk), and goats (product in milk). Data was collected from several articles,^16,95−99^ the Federal Drug Administration (FDA), and the European Medicines Agency (EMA) public databases. The figure is an update from Casteleijn and Richardson (2014).^100^

There is a clear trend in the past two decades in the choice of expression system toward mammalian host systems, and especially Chinese Hamster Ovary cells (CHO), over yeasts and bacteria to produce pharmaceutical proteins (Figure 2). The slight increase in *E. coli* products is partially due to the uptick of biosimilars’ market approvals, a growth market for biological drugs.^16^ In the beginning of the century, 20 pharmaceutical proteins were produced by transgenic technology for clinical trials.^95^ The high developmental costs for transgenic protein production at an industrial scale clearly hinder the advancement of this method,^95,96^ also seen by the approval of only four pharmaceutical proteins using this production method, outlined in Figure 2. Another problem for transgenic production arises during the development phase of the final product. The turnover from gene to production strain is much slower using animal hosts than when cells or cell-free systems. Here, mammalian systems are again slower than bacterial systems, while cell-free systems have the fastest turnover rate.

#### 3.1.1 *Escherichia coli*

The dominating organism of choice for recombinant protein production since the 1980s has been *E. coli.* Regarding pharmaceutical protein *E. coli* production, still accounts for 25% of marketed pharmaceutical proteins (Figure 2.), as such, it still is an important industrial host for many processes.^101^ The long history is also reflected in the understanding of *E. coli* genetics and the progress made in strain engineering for the production of proteins, plasmids and other molecules.^87,90,102−105^ Recent advances in glycosylation of heterologous proteins, the addition of disulfide bonds in both periplasmic and cytoplasmic space, and the expression of complex proteins highlight the importance of this production host for the production of pharmaceutical proteins.^106^

As such, *E. coli* is an excellent choice for the initial effort to produce a recombinant protein.^107^ A starting guide has been developed in the form of a consensus protocol for when little is known about whether the target protein can be recombinantly expressed in soluble and active form (i.e., expressibility).^108^ Another advantage is the culture conditions, which at large scale can be significant, since *E. coli* can be cultivated on relatively cheaply defined media (e.g., glucose, ammonia phosphate and some minerals) and strategies for low-cost production have been developed.^108,109^ On the other hand, due to its evident drawbacks, mammalian expression systems have advanced faster for the production of pharmaceutical proteins. These drawbacks include lack of suitable secretion systems and limited post translational modifications (PTMs; e.g., glycosylation is not trivial in bacterial systems). Moreover, *E. coli* produces pyrogenic endotoxins, although various methods can be employed for their removal,^110^ adding additional costs to the DSP.

#### 3.1.2 Yeast

Despite recent efforts to produce pharmaceutical proteins in *Kluyveromyces lactis* and *Yarrowia lipolytica*,^111^ only three other yeasts, *Hansenula polymorpha*, *Saccharomyces cerevisiae*, and *Komagataella pastori*, are currently utilized for the production of marketed pharmaceutical proteins (Figure 2). The major advantages for the use of these single-celled eukaryotic fungal organisms are stable production strains, durability, the possibility of high-density growth, high yield and productivity, rapid growth in chemically defined media, suitability for isotopically labeled protein production, their ability to glycosylate, the ability to assist protein folding, product processing that is similar to mammalian cell production, and the capability to handle multiple disulfide bonds formation in the target protein.^90,112^ Pharmaceutical proteins that cannot be produced in *E. coli* due to folding issues or proteins that require certain forms of glycosylation are often produced in yeast or mammalian cells.

*S. cerevisiae* has no pathogenic properties, and as such, it is classified as GRAS (generally regarded as safe). In general, *S. cerevisiae* is a good alternative to *E. coli*, also due to a comparatively well-characterized genome and well-established molecular biology tools. On the other hand, complex glycosylation patterns of the host organism are often undesirable for mammalian proteins due to O-linked oligosaccharides contain only mannose, whereas higher eukaryotic proteins have sialylated O-linked chains. Additionally, N-linked glycans are typically overglycosylated with high mannose type structures, which can lead to immunological responses and rapid clearance rates.^90,113^

The methylotrophic yeast *K. pastoris*, better known under its former name *Pichia pastoris*, is a versatile host for the expression of heterologous proteins for industrial purposes.^114,115^ One main reason for its use is the ease of applying well-established molecular biology tools developed for *S. cerevisiae*. As a host strain, it performs PTMs such as proteolytic processing, glycosylation, and disulfide bond formation quite well, with the additional benefit of glycoengineering.^116^ The expression system is available as a commercial kit; however, this may also be a hindrance for industrial uptake on large scales. High cell density cultivations in a bioreactor similar to *S. cerevisiae* and *E. coli* coupled with tightly regulated promoter systems, such as the AOX1^117^ and the SES system,^118^ has delivered protein titers at high level (intracellular or extracellular),^119,120^ however, as a host organism it also has its limitation when scaled up for industrial purposes.^121^ Low yields in particular cases can be attributed to poor transcription/translation.

Glycosylation is more restricted in *K. pastoris* than in *S. cerevisiae*.^122^ N-linked high-mannose oligosaccharides usually contain up to 20 residues. Similar to *S. cerevisiae*, glycoengineering has taken great strides in making humanized pharmaceutical proteins with regard to their glycan structures. Both human-like hybrid and complex N-glycans have been produced in *K. pastoris*.^123−125^

An in-depth review of *H. polymorpha* by Gotthard et al. highlighted its strengths for pharmaceutical protein production.^126^ A recent update of the field, focusing mainly on genetic aspects and fermentation protocols, concluded that *H. polymorpha* is still a promising host for the establishment of various bioprocesses.^127^ Similar to *K. pastoris*, proteins can be secreted into production media to simplify downstream processing. For secreted proteins, titers up to 13.5 g/L have been obtained (phytase).^126^ For pharmaceutical applications, the VP6 protein of rotavirus at 3.3 g/L and human serum albumin at 5.8 g/L are recent examples.^127^ The only marketed pharmaceutical protein produced by this host is recombinant HBsAg produced by Sanofi Pasteur, France.^16,128^ In *H. polymorpha*, N-linked oligosaccharides with high-mannose glycan chains are shorter than in *S. cerevisiae*. Typical oligosaccharide species are Man_8–12_ GlcNAc2-structures without terminal α-1,3-linked mannose residues. Therefore, the outer chain processing in the N-linked glycosylation pathway in *H. polymorpha* is similar to that in *K. pastoris*, with the lack of any terminal α-1,3-linked mannose residues and the addition of shorter mannose structures.

#### 3.1.3 Mammalian Cells, Including CHO

Up to 2023, over 60% of all pharmaceutical proteins are produced in mammalian cells (Figure 2), with the majority being CHO host cells strains.^16^ This reflects the well-known strengths of these production platforms, such as producing complex PTM and the ability to produce antibodies at titers of 3–8 g/L at production scale.^16^ The yields have been increased due to developments in bioprocess engineering, media optimization, and strain engineering since the 1980s.^129^ Despite the fact that adherent cell cultures can and are used in industrial production, the most abundant processes are developed for suspended cell cultures (e.g., CHO cell- and BKH cell-cultures), such as extended batch cultures and perfusion processes in clinical phase III-trials or during production phase.

For the manufacturing of pharmaceutical proteins that require complex PTMs, such as humanized glycosylation patterns, mammalian cell lines are the only viable option at relevant industrial scales, as the majority of therapeutic glycoproteins are produced in mammalian cells.^130^ However, the drawbacks to mammalian expression include the number of glycoforms that are expressed and the differences in protein glycosylation between different mammalian cell lines.^131^ Glycoproteins expressed in some production cell lines contain terminal N-glycolylneuraminic acid rather than human N-acetylneuraminic acid. This may affect immunogenicity, as antibodies against these nonhuman sialic acids moieties have been observed.^130^ Expression in some cell lines, such as human fibrosarcoma cell line HT-1080, can result in glycan chains with no terminal N-glycolylneuraminic acid moieties,^132^ however such alternative methods are not always possible.

Murine cell lines (e.g., NS0 and Sp2/0) produce glycan structures similar to those of humans, however they also produce immunogenic epitopes (e.g., Gal_α(1–3)_Gal). In addition, murine cell lines exhibit a high content of NeuGc sialic acid, which is why they are less commonly used for biotherapeutic production.^133^ One way to circumvent glycan structure problems, as well as other issues with PTMs such as disulfide bond formation, is to engineer cell lines to express proteins with the correct modifications.^134^ Due to their nonimmunogenic and near human-like glycosylation, CHO cells have become dominant in biotherapeutic production.^133,135^ Donini et al. provide a short but comprehensive overview of the advances in the field regarding pathway engineering and protein backbone engineering toward controlled and homogeneous glycosylation and novel glycan functionalities.^133^

#### 3.1.4 Outlook

Stably transfected CHO clones are the main expression systems for the development of recombinant pharmaceutical proteins. Transient gene expression, as a maturing technology,^136^ has due to its major recent advances,^137^ found approval for industrial pharmaceutical protein production (e.g., Luxturna (Spark Therapeutics; USA) and Zolgensma (Novartis Europharm; Ireland/Novartis Gene Therapies; (USA).^138^

Modern synthetic biology and post-transcriptional control (e.g., via CRISPR technologies or RNA aptamer–intramer fusions) will shed light on new expression strains: (i) as part of autologous cell therapies, gene circuits encode computational operations that can be programmed by intracellular signals to execute specific tasks, (ii) cell implants consisting of engineered allogeneic or xenogeneic mammalian cells could be plugged into the metabolism of patients to sense and respond to specific biomarkers.^139^ New advances in systems biology, machine learning, AI, and bioprocess optimization will accelerate the field.^140^

Alternative host systems for the production of pharmaceutical proteins are under investigation, for example the trypanosomatid protozoa *Leishmania tarentolae* (a nonpathogenic parasite) due to its complex PTMs and easier cultivation requirements than mammalian cells.^141−143^ In addition, the first clinical trials with a pharmaceutical protein, the C1-SARS-CoV-2 RBD vaccine, produced in the filamentous fungi *Thermothelomyces heterothallica*, have been concluded, proving the safety of alternative organisms.^144^ The main advances of this fungal host are high yields, advanced molecular biology tools available, and low cultivation costs compared to mammalian cells. In recent years, a CFPS system based on tobacco cells has emerged as a noteworthy development.^145,146^

### 3.2 Cell-Free Production of Pharmaceutical Proteins

The diversity of different cell species as production hosts for protein production can also be seen in the use of cell lysates derived from cells and subsequently applied in CFPS.

**Figure 3:**
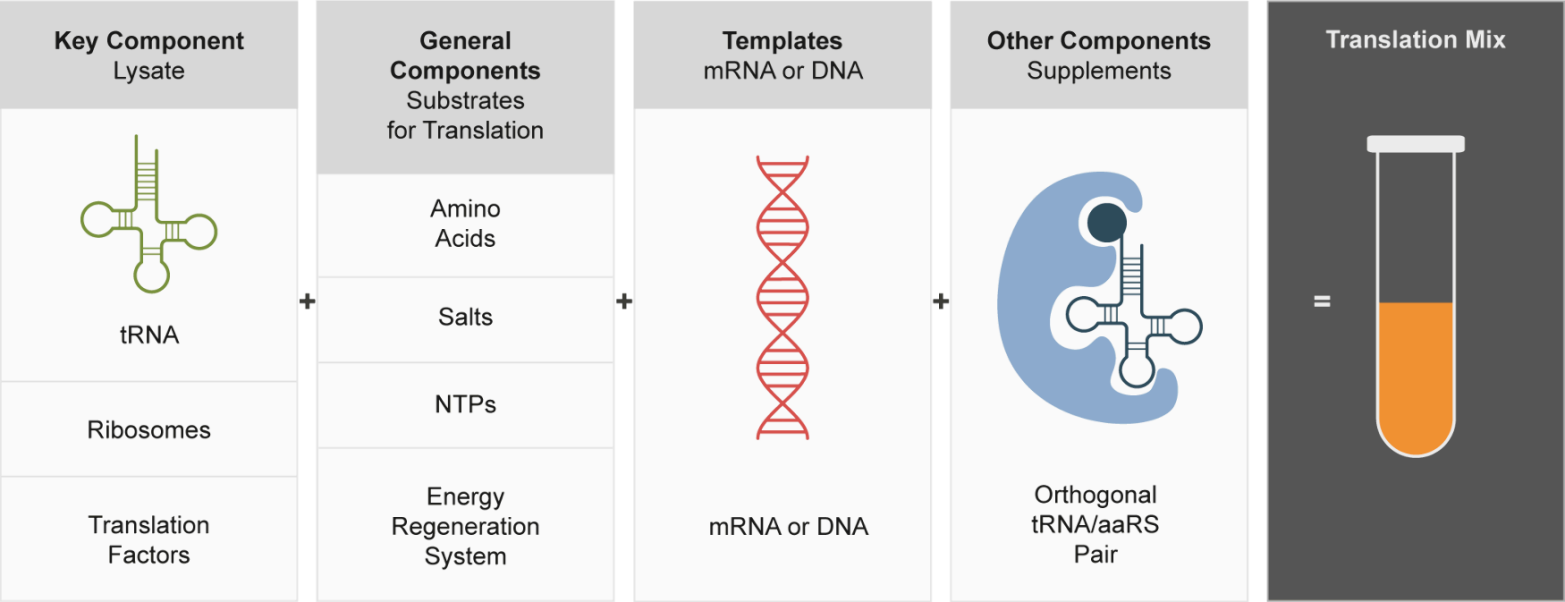
Schematic of cell-free protein synthesis. The key component of a CFPS reaction is usually a cell lysate containing the cellular translation machinery. Substrates for translation and other related processes like amino acids and NTPs are supplemented as well as a system for energy regeneration. RNA- (in uncoupled reactions) or DNA- (in coupled reactions) templates encoding the protein of interest are then added to induce protein synthesis. DNA templates can be added either as plasmids or as linear constructs. Due to the open nature of the cell-free reaction, reaction conditions such as pH and salt concentrations can be manipulated easily. Additionally other components such as tRNA/aaRS pairs for noncanonical amino acids (NCAA) incorporation, additional enzymes, chemicals for bioconjugation, and other cofactors can be added before or during the synthesis.

The foundation of CFPS was established in the 1960s by Matthaei and Nirenberg.^147^ The focus at that time was on studying the protein translation process in *E. coli.* Building on this research, various eukaryotic cell-free systems were developed, in addition to the previously mentioned prokaryotic system. These include protozoan, fungal (*S. cerevisiae*, *Komagataella phaffii* (*Pichia pastoris*)), plant (wheat germ, tobacco BY-2), insect (*Spodoptera frugiperda* 21), and mammalian (rabbit reticulocytes, CHO, K562, HEK293, HeLa) based cell-free systems.^148^ Despite their different origins, each system is based on translationally active cell lysates, which contain the complete translation machinery, thus enabling protein synthesis. For the production of cell lysates, selected cell lines are fermented and lysed at a defined cell density. While the nucleus, cellular debris, and endogenous mRNA (mRNA) are removed, essential components for protein synthesis such as ribosomes, aminoacyl-tRNA synthetases, translation factors, and chaperones are retained.^149^ The resulting cell extract is then supplemented with energy in the form of ATP and GTP, an energy regeneration system, and amino acids. The addition of the nucleic acid template can be in the form of circular or linear DNA (“coupled”) or as pretranscribed mRNA (linked or uncoupled).^150^ In the linked mode, translation is separated from transcription by a gel filtration step, which allows the setting of optimal parameters such as temperature, buffer conditions, and salt concentration (Figure 3).^151^ Typical protein yields of common CFPS systems are listed in Table 3.

**Table 3:** Protein Yields of Commonly Used Cell-Free Systems Used for the Synthesis of Pharmaceutical Relevant Proteins

System	Yield [μg/mL]	Synthesized protein	Ref
*E. coli*	2300	GFP	Caschera et al. 2014^152^
	1400	Trastuzumab and brentuximab	Groff et al. 2014^191^
	700	GM-CSF	Zawada et al. 2011^156^
*V. natrigens*	250	Opistoporin	Des Soye et al. 2018^192^
*L. tarentolae*	300	eGFP	Mureev et al. 2009^193^
Wheat germ	1600	GFP	Harbers 2014^194^
	>20	scFv	Kawasaki et al. 2003^195^
Tobacco	3000	eYFP	Das Gupta et al. 2022^146^
	150	vitronectin-specific full-size antibody M12 CNTF	Buntru et al. 2015^196^
	20		Richardson et al. 2018^48^
*S. cerevisiae*	60	HPV-VLP	Wang et al. 2008^197^
	40	EPO	Sullivan et al. 2016^198^
Insect (*Sf*21)	286	EGFR	Quast et al. 2016^199^
	30	Anti-FITC scFv	Stech et al. 2013^179^
CHO	950	EGFR	Thoring et al. 2017^175^
	250	IgG and scFv-Fc	Stech et al. 2017^200^
	124	EPO	Gurramkonda et al. 2015^176^
Human	43	Hirudin	Wüstenhagen et al. 2020^184^
	49	CNTF	Richardson et al. 2018^48^

Similar to heterologous protein expression, *E. coli* continues to be the predominant system for CFPS (Figure 3). After decades of optimization, *E. coli* CFPS routinely achieves yields of target proteins exceeding 1 mg/mL.^152,153^ Its versatility is evident in the synthesis of various antibody formats and other pharmacological proteins such as Serratiopeptidase^154^ and antimicrobial proteins like colicins.^155^ The scalability of *E. coli* CFPS has been demonstrated, with reactions scaled up to 100 L, as exemplified in a study showcasing the production of GM-CSF.^156^ However, the most promising applications lie in personalized medicine and the point-of-care synthesis of pharmaceutical products, given the minimal requirements for conducting a cell-free reaction.^157^ Additionally, CFPS offers screening capabilities, e.g., facilitating the rapid development and identification of new antibodies.^158^ Similar to cell-based approaches, synthesizing more complex eukaryotic proteins in *E. coli* CFPS can pose challenges. Despite numerous attempts to enhance disulfide bond formation^159^ and the incorporation of specific glycans,^152,153,160−162^ the correct folding and post-translational modification of these proteins remain limiting factors.

While yeasts are extensively employed in industrial large-scale production, their utilization for CFPS is relatively underdeveloped. Interestingly, a huge effort was made by Jewett and co-workers starting in 2013 to generate a highly productive cell-free system based on *S. cerevisiae*.^163^ They optimized different factors such as the extract preparation, byproduct removal, energy metabolism and implementation of an internal ribosome entry site (IRES).^163−166^ The IRES element, discovered in the 5′ untranslated region of mRNAs, enables translation initiation in a cap-independent manner, adding flexibility for utilizing CFPS.^167^ Finally, they were able to utilize their developed system for a yeast-based ribosome display method to evolve cap-independent translation initiation sequences.^168^ Furthermore, Jewett and co-workers were able to create a knockout library for rapidly prototyping strains for cell-free protein synthesis.^169^ This idea was taken up later on by Polizzi and co-workers who undertook strain engineering of *P. pastoris* to increase protein production efficiency.^170^ With this approach, they reached a remarkable titer of 48.1 mg/L human serum albumin. Polizzi et al. further pushed the dissemination of protocols for the development of yeast-based cell-free protein synthesis systems.^171^ In recent research on CFPS-based yeast systems the focus is still on strain engineering for cell-free biomanufacturing^172^ and translation mechanism analysis.^173^ Systems involving *S. cerevisiae* and *P. pastoris* have been demonstrated to function but still have an acceptance level that falls behind other eukaryotic cell-free systems.

The dominance of CHO as a mammalian expression host also has an impact in the area of CFPS, as it is the highest-yielding mammalian CFPS system reported so far, reaching 500 mg/L and above for some target proteins.^174,175^ Due to the presence of ER-derived membranous structures, the synthesis of membrane proteins and incorporation of PTMs like glycans are supported. This enables the synthesis of pharmaceutically relevant glycoproteins, such as EPO.^176^ The PEGylated form of EPO (Mircera) was already approved by the FDA in 2007 and is prescribed against anemia associated with kidney diseases.^177^ Since it is known that the human body can form anti-PEG antibodies and that PEG can therefore trigger allergic reactions and limit the effect of the therapeutic agent, research has been carried out into alternative stabilizers to PEG. Such an alternative could be based on linear polyglycerol (LPG) due to its similar structure and characteristics compared to PEG.^178^ A first comparison of the cell proliferation effects of LPG- EPO and PEG-EPO was performed by synthesizing the different molecules in an insect-based cell-free system. The LPG-EPO showed a comparable activity and demonstrated a prolonged half lifetime compared to nonmodified EPO.^62^

Other examples include various antibody formats^179−181^ and bone morphogenetic protein.^182^ In the latter example, the synthesis yield of human bone morphogenetic protein was compared in cell-free and cell-based expression system. Interestingly, the CHO-based cell-free synthesis was able to produce a much higher protein yield (40 μg/mL) compared to stably transfected CHO cells (153 pg/mL) and transiently transfected HEK cells (280 ng/mL). The limited yields in cell-based expression systems might result from a negative feedback interaction of the synthesized protein.^183^

Beside CHO, also human cell extracts can be utilized to perform CFPS. However, they usually lag in terms of protein yields compared to the CHO-system.^184^ Recently Aleksashin et al. described a highly efficient human cell-free translation system based on HEK293T cells.^185^ They reinvented the work of Mikami et al.^186^ by also increasing the amount of GADD34, thus improving transcription. They improved translation efficiency by engineering cells to endogenously express GADD34 and K3L proteins, which suppress phosphorylation of translation initiation factor eIF2α. With this adaptation, they were able to get a 30-fold increase of active Nluc expression.^185^ Unfortunately, the total protein yield or final concentration is not mentioned in the publication.

*L. tarentolae* is also used for CFPS.^187,188^ Besides the first optimization studies only limited reports are based on *L. tarentolae*. Quite recently, *L. tarentolae* was used in comparison to an *E. coli* cell-free system to develop a rapid and cost-effective polypeptide prototyping system. With this system, a wide variety of disulfide-constrained peptides, macrocyclic peptides, and antibody fragments were successfully synthesized in an active form.^189^

While initially, the utilization of a plant-based system for the production of human proteins seems to have some drawbacks, initial reports on the tobacco BY-2 system indicate promising prospects. With yields reaching up to the mg range and its scalability, allowing for reaction volumes up to 10 L, it currently stands as the most productive eukaryotic CFPS system.^146^ Remarkably, it surpasses the more prominent representative of plant-based CFPS, wheat germ extract, in terms of both efficiency and productivity. Similar to mammalian lysates, tobacco lysates contain microsomes, facilitating the translocation and post-translational modification of target proteins. This capability enables the synthesis of pharmaceuticals such as full-length antibodies, epidermal growth factor (EGF)^146^ and virus-like particles.^190^

Since mammalian cells, *E. coli* and yeast cells are the most common expression systems for *in vivo* (Figure 2) and *in vitro* expression of proteins, we briefly consider the cost of CFPS. For example, regarding *E. coli*, in 2012 the cell-free transcription-translation (TXTL) system was compared to other *E. coli* systems and can therefore be seen as a relative measure. The cost per mg protein was estimated to be $ 4.00/mg,^201^ an equivalent in purchasing power to about $5.46 today.^202^ It is due to the lower cost of *E. coli* CFPS that this system has been applied at larger scale, e.g., by Sutro Biopharma.^93,156^ As such, bioconjugated pharmaceutical proteins can be produced at reasonable cost at small scale, e.g., the *E.* coli-based polysaccharide-protein conjugate system (iVAX), using protein glycan coupling technology, can produce 24 μg of conjugate vaccine (≃ 1 dose) for $ 0.50 - $1.00 per dose depending on storage conditions. In contrast, *in vivo* production in *E. coli* in optimized bioprocesses costs $0.04/g, which is several orders of magnitude lower.

With increasingly reported higher yields for *K. pastoris*, the production costs per mg of protein are being reduced;^170,203^ however, yields of *S. cerevisiae* are still relatively low.

Regarding mammalian CFPS, a techno-economic assessment (TEA) revealed the cost differences between small-scale production (up to 25 kg of mAb/year) and large-scale production (up to 200 kg of mAb/year) of CFPS versus CHO cell-based production.^204^ DNA recycling was discovered as a significant cost-reducing factor for CFPS. For large scale production, the unit production cost (UPC) for *in vivo* production is $85, while for *in vitro* production, the cost is $1925. In the smaller-scale CFPS the UPC was in the same order of magnitude, $986 (*in vivo*) versus $2466 (*in vitro*). Several suggestions were made to reduce the cost of CFPS; however, the highest costs were related to operational costs, DNA concentrations, and enzyme amounts needed in the reaction.

It is obvious from these few examples that if rapid development is a significant cost factor, then CFPS could potentially be important for production at scale. In addition, due to the open nature of CFPS, pharmaceutical protein development, especially when combined with bioconjugation, could become competitive in the future. However, for simple proteins, such as industrial enzymes or for food production, CFPS must solve the increased production to titers well over 20–50 g/L, while reducing the material costs.

Antibody-based drugs have emerged throughout the past few decades as the most important class of pharmaceutical proteins. The first full-length IgG (mouse monoclonal antibody MAK33) was successfully synthesized by Frey et al. in 2008 using an *E. coli* cell-free system supplemented with protein disulfide isomerases and chaperones.^205^ Four years later, Yin et al. demonstrated for the first time the successful synthesis of the therapeutic antibody trastuzumab. Moreover, they were able to conduct the synthesis in a scalable transcription/translation system with protein yields of ∼400 μg/mL.^206^ Ever since the first antibody was produced in a CFPS system,^207^ the field has moved rapidly to include more complex antibody-conjugated protein drugs.^207^

The described examples of cell-free synthesized ADCs were done in prokaryotic-based cell-free systems with limited posttranslational modifications such as glycosylations and limitations in folding and assembly of full-length IgG. For some antibodies, glycosylation is crucial for conformation and stability.^208^ Therefore, the use of eukaryotic systems could be beneficial. In particular, systems that contain endogenous membrane vesicles based on endoplasmic reticulum, so-called microsomes can perform core glycosylation and disulfide bridging.^209^

The synthesis of full-length IgG has been demonstrated in different eukaryotic systems. For example, Buntru et al. produced a vitronectin-specific full-size human antibody in a tobacco BY-2 cell lysate by coexpressing the HC and LC by two different vectors.^196^ By enriching the BY-2 lysate with an 8-fold amount of microsomes, the total protein yield of the antibody was increased 4-fold up to 150 μg/mL. The BY-2 lysate has been further evolved in recent years to serve as a production platform.^145^ Adalimumab was synthesized at 10 mL scale showing comparable binding affinities to CHO-produced mAb.^210^

Not long thereafter, two research groups independently demonstrated the successful synthesis of an IgG in CHO cell-free systems. Martin et al. used a commercially available CHO extract and optimized the reaction conditions by establishing an oxidizing environment to maximize protein yield of disulfide bridged antibody.^181^ This system was utilized as a tool for ranking the yields of candidate antibodies for automated expression analysis. In contrast, Stech et al. have used an in-house produced CHO cell-free system with endogenous microsomes for the synthesis of a SMAD2 antibody.^200^ The adaptation of the reaction conditions to an oxidizing environment was not necessary for this construct.

In addition to the synthesis of complex proteins, CFPS facilitates the synthesis of peptides. Using the parallelizability of the system, CFPS becomes a valuable tool for screening biologically active peptides, such as antimicrobial peptides.^211,212^ Furthermore, ribosomally synthesized post-translationally modified peptides (RIPPs) are promising candidates for pharmaceutical applications, including antitumor agents and antimicrobial ingredients. However, achieving these modifications in cell-free systems involves the recreation of biosynthetic pathways, as exemplified by lanthipeptides like the antimicrobial Nisin.^213^ In a different approach, various biosynthetic clusters for the synthesis of lasso peptides were expressed cell-free, concurrently with a library of fewer than 1000 template sequences, resulting in a diverse array of newly sequenced lasso peptides.^214^ Expanding further, CFPS has been employed to synthesize entire nonribosomal peptide synthetase (NRPS) complexes. Pioneering this effort, Goering et al. synthesized two NRPS complexes, each exceeding 100 kDa, enabling the subsequent synthesis of the precursor molecule diketopiperazine.^215^ Recently, a groundbreaking achievement was demonstrated with the extract-based cell-free synthesis of a final natural product using valinomycin as an example. This was accomplished through the one-pot synthesis of two complete NRPSs, 370 and 284 kDa, respectively. Further process optimizations and a switch to a two-step reaction led to yields of 30 mg/L of valinomycin.^216^ With several cell-free produced biologics currently in clinical trials, all the way up to phase III, it is only a matter of time before we see true industrial applications come to market.

## 4 Co-translational Incorporation of Noncanonical Amino Acids

The use of noncanonical amino acids (NCAAs) is an important method to introduce unique chemical handles, e.g., azide, aldehyde, or ketone, by replacing a natural amino acid with its analogue. Bioorthogonal groups are strategically positioned in the protein to have a minimal effect on the conformation of the target-binding site to avoid interference with the protein’s activity.^217^

**Figure 4:**
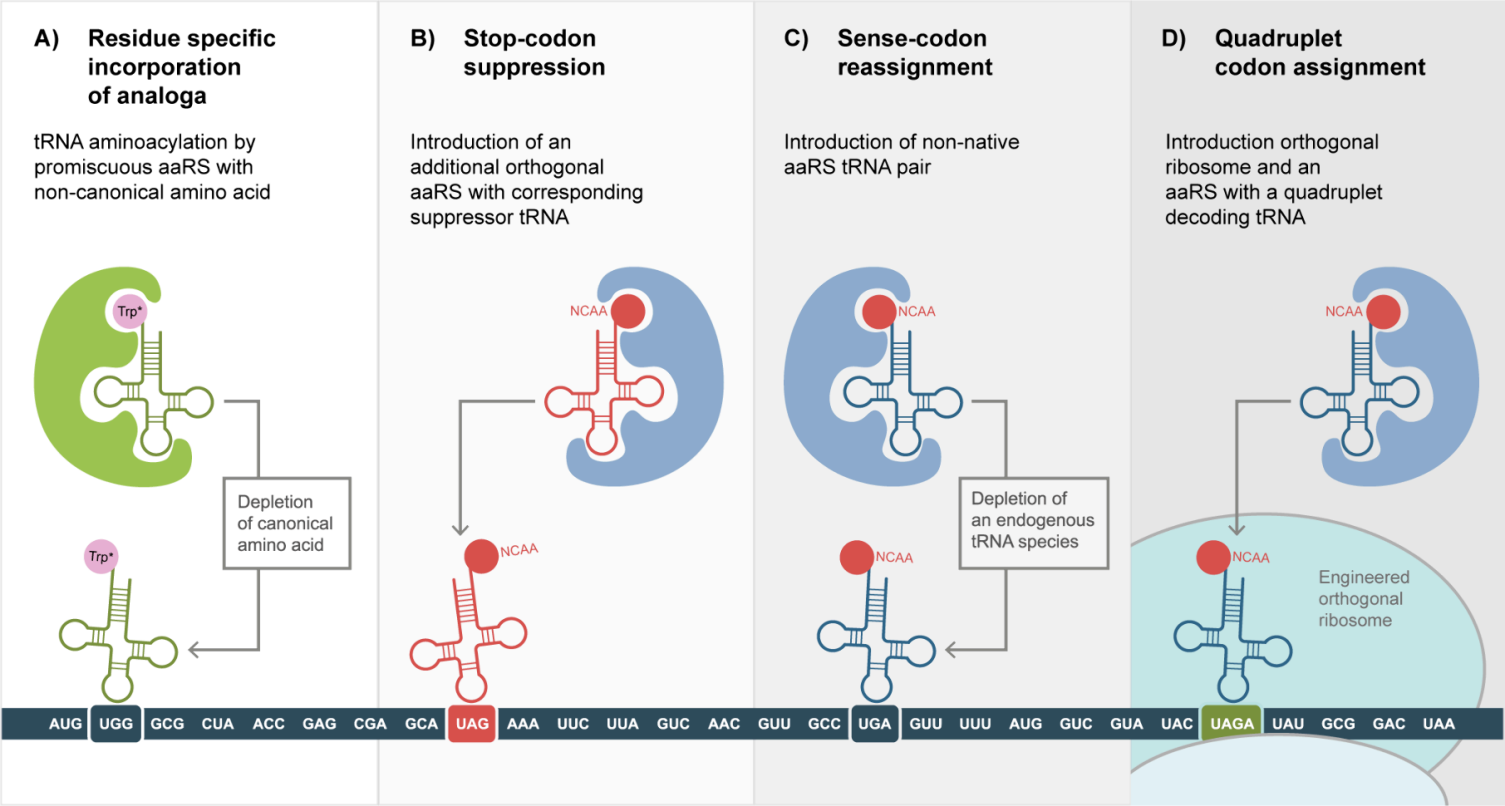
Strategies for cotranslational incorporation of noncanonical amino acids. A) Depletion of a canonical amino acid from the growth media or cell-free reaction mix with the simultaneous supplementation of a noncanonical analogue can lead to an incorporation of the analogue instead of the original amino acid. This however, requires the corresponding aaRS to have a certain promiscuity toward its substrate, either be default or through protein engineering. This results in a protein wide replacement of the canonical amino acid by its analogon incorporation. B) Utilizing a tRNA that recognizes a stop-codon, the stop-codon can be suppressed to incorporate a NCAA. Recharging of the tRNA can be realized by the addition of a corresponding aaRS. To ensure site-specific NCAA incorporation at the stop-codon position, the tRNA and aaRS pair must be orthogonal to the host system, meaning that there is no inteference between endogenous tRNA/aaRS and the newly introduced pair. C) By depleting or deleting certain endogenous tRNA species, vacant codons are created. These codons can be reassigned to the NCAA using orthogonal tRNA/aaRS pairs. D) By the introduction of quadruplet codons the genetic code can be expanded further; this requires tRNAs recognizing these quadruplets specifically as well as corresponding orthogonal aaRS. Together with the use of orthogonal mRNAs and ribosome, this enables the introduction of several new codons in one template.

Orthogonal protein translation with NCAAshas become a common method in biosciences. Even though many endeavors are made to broaden the NCAA’s chemical space, much work is still to be done regarding their systematic, low-cost *in situ* production (Figure 4). Improved host cell strains need to be engineered to utilize designed biosynthetic pathways coupled with orthogonal aminoacyl-tRNA synthetase/tRNA pairs (o-pairs). These host strains are needed to provide cost-effective solutions for industrially relevant pharmaceutical proteins. Therefore, coupling genetic code expansion (GCE) with metabolic engineering is the basic prerequisite to transform orthogonal translation from a standard technique in academic research to industrial biotechnology.^218^

From a historical perspective, the utilization of NCAAs in the integration of proteins and peptides represents a prevalent strategy to broaden the functional repertoire of these biomolecules. Various techniques have been employed to accomplish this objective. Approaches for incorporating NCAAs into proteins can be grouped into two principal categories: cotranslational and post-translational strategies. While post-translational modifications surely have their advantages, this review centers on the cotranslational methods.

The advent of chemical^219^ and chemoenzymatic methodologies^219,220^ for aminoacylation of tRNA introduced the possibility of misacylation of tRNAs. This paved the way for enabling the site-specific integration of noncanonical amino acids by conjugating them to a suppressor tRNA specific to the amber stop codon (UAG).^221,222^ Propelled by novel discoveries like the Flexizyme technology, which facilitates tRNA aminoacylation through a ribozyme,^223^ this approach can serve as a readily available solution for protein modification. A key advantage lies in its substrate flexibility, as it is not constrained by the specificity of aminoacyl-tRNA synthetases, enabling the incorporation of diverse bulky NCAAs, such as conjugated fluorophores.^224^ However, even within synthetic cell-free systems, a limitation persists concerning the size of NCAAs, dictated by tRNA recognition by the elongation factor EF-Tu (eEF-1 in eukaryotes).^225,226^ Nevertheless, the primary drawbacks are the constrained yields and the restricted applicability to protein expression in live cells, as exemplified by the microelectroporation of CHO cells with tRNA.^227^

Ohno et al. utilized a yeast aminoacyl-tRNA synthetase (aaRS) and tRNA (tRNA) pair in *Escherichia coli* to facilitate amber suppression, marking a significant milestone as the first aaRS capable of charging a suppressor tRNA in 1998.^228^ The initial orthogonal aaRS systems employed for NCAA incorporation were derived from a tyrosyl-tRNA synthetase (TyrRS), as described by Wang et al.^229^ and Chin et al.^230^ The first orthogonal TyrRS system tailored for the incorporation of O-methyl-l-tyrosine based on the TyrRS/TyrT from the archaea *Methanococcus jannaschii* (*mj*TyrRS/*mj*TyrT) exhibited orthogonality in *E. coli*(229) but not in mammalian cells.^231^ A limitation that can be circumvented by using an alternative pair based on the *E. coli* TyrRS/TyrT, allowing NCAA incorporation in mammalian systems.^230^ Additionally, a hybrid of these two synthetases was designed to combine the functionality of *mj*TyrRS with the orthogonality of *ec*TyrRS toward mammalian cells.^232^ To this day, TyrRS-based orthogonal pairs continue to be extensively used, enabling the incorporation of more than 50 predominantly aromatic NCAAs.^233^

Orthogonal pairs based on other canonical aminoacyl-tRNA synthetases, such as Tryptophanyl-tRNA synthetase (TrpRS)^234^ or Leucyl-tRNA synthetase (LeuRS)^235,236^ have also been reported but are less commonly employed.^237^ However, the most prominent orthogonal pairs originate from the archaeal PylRS family. These naturally occurring synthetases were initially discovered in 2002 within methanotrophic archaea, where they facilitate the integration of pyrrolysine into nascent proteins. A notable characteristic is their natural capability as amber suppressors.^238^ Moreover, PylRS lacks an anticodon-recognition domain and does not rely on anticodon recognition by tRNA,^239,240^ making it suitable for opal (UGA) and ochre (UAA) suppression.^241,242^ Due to their archaeal origin, they naturally exhibit orthogonality in both prokaryotes and eukaryotes,^243^ with yeast being the exception.^244^

Alternatively, an approach to enable the incorporation of multiple NCAAs involves the addressing of sense codons. One straightforward method to achieve this is by capitalizing on the promiscuity of certain aaRS. This can be accomplished, for instance, by feeding a *E. coli* strain with an auxotrophy for a specific amino acid, with an analogue of just that amino acid. This was first shown by growing a leucine auxotroph strain in a leucine-depleted medium supplemented with the leucine analogue, 5′,5′,5′-trifluoroleucine.^245^ Strategies of this nature can find utility in incorporating labeled NCAAs to assist in structural elucidation via X-ray crystallography and NMR,^246^ or enhancing protein stability.^247^ Such endeavors can benefit from further engineering of the specific aaRS to enhance their acceptance of the analogue.^248^ This approach can also be applied to introduce multiple NCAAs.^249^ However, it is important to note that this method lacks specificity and there is a possibility of adverse effects resulting from protein-wide NCAA incorporation.

A more precise approach involves the reassignment of sense codons, necessitating the construction of vacant codons through genomewide substitution with synonymous codons. This was exemplified in *E. coli*, where 62,214 codons were replaced, resulting in an *E. coli* strain with only 57 codons.^250^ In another strategy, a 61-codon *E. coli* strain was designed, leaving the amber codon and serine codons (TCG and TCA) vacant.^251^ Further refinement of this strain has facilitated the incorporation of various NCAAs into GFP multimers.^252^

Taking translational machinery redesign to the next level, quadruplet codons have been harnessed for the incorporation of NCAAs.^253^ Building upon the foundation of an orthogonal ribosome designed to decode amber codons from an orthogonal mRNA,^254^ orthogonal ribosomes have been engineered to decode quadruplet codons, thereby enabling the integration of NCAAs.^255^ This pioneering work soon found its applicability in mammalian cells,^256^ and ongoing refinements^257−259^ have resulted in increased efficiency, allowing for the incorporation of up to four distinct NCAAs in *E. coli*.^258^

Though the protein yields from such technologies are currently economically impractical, they are pushing the boundaries of life itself, providing a glimpse into the future of synthetic biology. At the same time, we have access to several well-established and robust methods today that facilitate the incorporation of over 200 structurally diverse NCAAs.^233^ The applications of these methods range from protein labeling,^260,261^ incorporation of a variety of PTMs,^262^ supporting live-cell^263^ and super-resolution imaging,^264^ to expanding the genetic code of living multicellular organisms themselves,^265,266^ even treating diabetes in mice.^262^

### 4.1 The Role of tRNAs and tRNA Modifications in CFPS and NCAA Incorporation

Transfer RNAs play a crucial role in the intricate process of translation, serving as molecular adaptors that bridge the genetic information encoded in mRNA with the amino acid sequence of proteins. These small RNA molecules, typically consisting of about 70–90 nucleotides, are essential components of the cellular machinery responsible for protein synthesis.^267^ Each tRNA molecule is charged with a specific amino acid, and during translation, it accurately interprets the genetic code by base-pairing with the complementary codon on the mRNA. This critical interaction ensures the correct placement of amino acids in the growing polypeptide chain, facilitating the precise translation of the genetic information from nucleic acids to functional proteins.^268^ The adaptability and specificity of tRNAs in recognizing both codons and amino acids make them fundamental players in translation.^269^ Although tRNAs are initially transcribed from genomic DNA, they undergo a series of modifications, such as for example methylation and thiolation, which are crucial for their structural stability, accurate decoding of mRNA codons, and participation in the translation process.^270−272^ These post-transcriptional modifications of tRNAs play a pivotal role in ensuring their optimal functionality during protein synthesis. The modifications influence tRNA folding, stability, and interactions with aminoacyl-tRNA synthetases, ensuring proper amino acid charging.^273^ To date, 334 different nucleoside and nucleotide modifications are reported.^274^ Additionally, modified bases within the anticodon region contribute to codon–anticodon recognition, enhancing the fidelity of translation.^275^ The diversity of post-transcriptional modifications not only enhances the overall structural integrity of tRNAs but also fine-tunes their binding properties. *E. coli* tRNAs harbor up to eight modifications in one tRNA meaning that approximately 12% of the nucleosides of the molecules have additional modifications.^276^ In summary, post-transcriptional modifications are essential for maintaining the functionality and accuracy of tRNAs, ultimately impacting the precision and efficiency of protein synthesis in cells.^277^

In CFPS systems, tRNAs play a crucial role as essential mediators of translation. CFPS allows for protein production outside living cells by utilizing purified components of the translation machinery. tRNAs, charged with specific amino acids, serve as key adapters in the decoding process. They accurately recognize and pair with codons on the mRNA template, facilitating the incorporation of amino acids into the growing polypeptide chain.^268^ The manipulation of tRNAs and tRNA populations holds significant potential in CFPS, offering a versatile avenue for tailoring protein production. By introducing engineered tRNAs with altered specificity or charging capabilities, researchers can expand the repertoire of amino acids that can be incorporated into proteins, thereby enabling the synthesis of proteins with diverse chemical functionalities.^278,279^ Additionally, tRNA manipulation in CFPS systems provides a means to optimize translation efficiency, fine-tune codon usage, and enhance the fidelity of protein synthesis. This level of control is particularly advantageous in the context of CFPS, where reactions can be tailored for specific applications, such as the production of modified or labeled proteins for structural studies, biotechnological applications, or even the creation of artificial, biobased materials.^280,281^ Overall, the ability to manipulate tRNAs in CFPS opens avenues for innovative and customized protein synthesis strategies. Researchers can achieve precise control over protein synthesis, offering a versatile platform for protein engineering and synthesis in a controlled laboratory setting.

### 4.2 Common Approaches of tRNA Manipulation in CFPS

#### 4.2.1 Stop-Codon Suppression

Stop codon suppression is a naturally occurring process in certain organisms that exhibit an expanded genetic code to incorporate selected amino acids in response to a stop codon. For example, selenocysteine is encoded by the opal codon and pyrrolysine by the amber codon.^282,283^ Stop codon suppression is widely used for site-directed incorporation of NCAAs into proteins and is achieved by the introduction of the stop codon by site-directed mutagenesis. However, due to competition between the release factors and the suppressor tRNA for interaction with the stop codon, usually two types of translation products are obtained: the truncated termination product and the full-length suppression product containing the desired NCAA. Recent research, aimed at enhancing the efficiency of incorporating NCAAs into proteins and facilitating the incorporation of multiple copies of an NCAA, has primarily concentrated on methods to minimize interference with release factors. In *E. coli*, RF1 is targeting TAG (amber stop codon), and RF2 is targeting TGA (opal stop codon), with both release factors recognizing TAA (ochre stop codon).^284^ In *E. coli* RF1 has been successfully deleted to increase the incorporation of NCAA.^285^ Further development of this strain was done by large-scale mutagenesis of the TAG stop codon to TAA to minimize the readthrough of endogenous stops and suppress the negative side-effects of the RF1 deletion. Mukai et al. exchanged 95 TAG stop codons in an *E. coli* BL21(DE3) RF1 deletion strain showing that the growth defect of the RF1 deletion could be rescued.^286^

#### 4.2.2 Sense Codon Reassignment

There are 61 naturally occurring sense codons with a great deal of redundancy, as groups of two to four and, at best, even six codons are synonymously read by families of tRNA isoacceptors. This degenerated code allows for the reassignment of sense codons. Sense codon reassignment is a process that involves replacing one or more sense codons in the genetic code, followed by the removal of the decoding tRNA. This frees up the codon from the canonical genetic code and allows for the reassignment of the codon to encode an NCAA. This process is termed synonymous codon compression. The pioneer work in establishing the concept of genetic code reprogramming by sense codon reassignment was done by Forster et al. The authors reassigned three sense codons to the ochre codon UAA using chemoenzymatically charged tRNAs in a reconstituted translation system lacking aaRSs.^287^ Recent advances elucidate the role of tRNA modifications in enhancing sense codon reassignment.

Queuosine is a nonessential, hypermodified guanosine nucleoside found in position 34 of the anticodons of four *E. coli* tRNAs. One suggested purpose of queuosine at position 34 is to reduce the preference of tRNAs with guanosine at position 34 of the anticodon for decoding cytosine-ending codons over uridine-ending codons. Queuosine modification has been identified in tRNAs having QUN anticodons across most organisms.^288^ Furthermore, m1G37 modification in tRNA CGGPro of *E. coli* is required for high-affinity binding to a cognate CCG codon in the decoding center of the ribosome. The m1G37 modification in anticodon stem loop stabilizes high-affinity interactions in the cognate case but prevents recognition of slippery codons that would result in −1 frameshifting.^289^

#### 4.2.3 Synthetic tRNAs in PURE

Synthetic tRNAs are an option to introduce reassigned tRNAs back into the CFPS reaction. An outstanding example is the *E. coli* PURE system, which stands for “Protein synthesis Using Recombinant Elements”, and is a well-established reconstructed CFPS platform that enables the *in vitro* synthesis of proteins using purified components derived from *E. coli*. This system provides a controlled and defined environment for protein production, allowing researchers to study and manipulate translation processes outside the complexities of living cell. Enzymes involved in transcription and translation, such as RNA polymerase and aminoacyl-tRNA synthetases, are recombinantly produced and purified. This allows for the efficient initiation and elongation of protein synthesis. The drawback of the synthetic or *in vitro* transcribed tRNAs is the lower fidelity and drop in overall protein yield. In fact, new advances by McFeely et al. (2022) demonstrated the superior performance of fully modified wildtype tRNAs over the t7 tRNA in encoding multiple NCAA within a single codon box.^290^ The 6-fold degenerate leucine codon family can be reassigned to encode three amino acids, including two NCAAs. The wild-type tRNA, but not the *In vitro* transcribed tRNA, was discriminated with enough fidelity to support the biosynthesis of a peptide bearing two NCAAs in a PURE translation system.^291^

### 4.3 tRNA Capture Techniques for Depletion

To allow the specific application of synthetic tRNAs, CFPS extracts need to be depleted from the native tRNA pools. The depletion of the total tRNA pools is achieved by using two commonly used techniques:

1. Ethanolamine Sepharose columns: It was discovered by accident that 90% of native tRNA in rabbit reticulocyte lysates could be separated by covalent interactions using the chemical groups of ethanolamine anchored to the resin.^292^ For this method, a column of epoxy-activated Sepharose 6B is used. Optimization of the equilibration buffer of the column resulted in the elimination of about 95% of the total endogenous tRNAs in S30 extracts.^293^ Although the process is simple and the removal efficient a small amount of tRNA is still present, which can interfere with reassigning the genetic code.
2. RNase-coated magnetic beads: This approach utilizes superparamagnetic beads coated with ribonuclease A (RNase A) to enzymatically degrade tRNAs within the cell extract. The activity of the RNase A attached to the beads can be regulated to degrade tRNAs, and subsequently, the RNase A can be removed from the extract. This protocol makes full use of the protective effect of nucleoproteins, meaning that the RNase A degrades tRNAs but not rRNAs which are in complex with ribosomal proteins.^294^ Additionally, the cell extract is treated with phenylmethylsulfonyl fluoride (PMSF) to inhibit proteases and prevent leaching of RNase A into the cell extract. The effectiveness of tRNA removal was demonstrated with an average removal ratio of 99.3% after 60 min of incubation.

Other approaches include the substitution of specific tRNAs to facilitate the tRNA reassignment. For this, only a subset of the tRNAs is removed from the cell extract. Here, resin-bound colicin D and DNA hybridization chromatography have been successfully used.^295−299^

### 4.4 tRNA Purification Methods

Several methods are available for the purification of specific individual tRNAs from *in vivo* environments. These include the hydrophobic tagging method, DNA probe-elution method, and DNA probe-digestion method.^300−304^ The hydrophobic tagging method involves using a hydrophobic tag to isolate and purify specific aminoacylated tRNAs based on their high molecular weight. The DNA probe-elution method utilizes biotinylated DNA oligonucleotides immobilized on streptavidin agarose beads to isolate individual tRNAs from total RNA. Lastly, the DNA probe-digestion method involves the use of biotinylated DNA oligonucleotide probes to extract targeted tRNA fractions, which are then released via digestion with DNase I. On the other hand, *in vitro* tRNA production methods include enzymatic and chemical synthesis.^305,306^ Enzymatic synthesis involves using T7 RNA polymerase to transcribe tRNAs. However, the transcription efficiency of T7 RNA polymerase depends on the specific recognition of its cognate promoter sequence, which can result in 3′-end heterogeneity.^307^ Chemical synthesis involves solid-phase chemical synthesis, which allows for modifications and easy purification but requires expensive equipment.^308^

The addition of purified tRNAs circumvents limited and species dependent codon usage during protein synthesis. This involves addressing the redundancy in the standard genetic code by excluding the influence of endogenous tRNAs in a cell-free system. The tRNA-depleted S30 extract and PURE ΔtRNA system have been used for reassigning sense codons in protein synthesis, allowing for the construction of a tRNA pool covering the decoding of 20 natural amino acids.^309,310^ Although challenges remain in completely removing native tRNAs, this approach has significantly broadened the artificially designed platform for protein synthesis using the smallest number of codons and allowed for the incorporation of NCAAs.

#### 4.4.1 Future Developments

Efforts to engineer the binding pocket have led to the incorporation of over 100 different chemical moieties by PylRS from *Methanosarcina barkeri* (*mb*PylRS) and *Methanosarcina mazei* (*mm*PylRS).^233^ However, their principal limitation lies in the N-terminal domain’s propensity for aggregation, which can potentially be mitigated with N-terminal solubility tags.^311^ Genome mining efforts have unveiled a novel type of PylRS from *Methanomethylophilus alvus* lacking the problematic N-terminal domain.^312^ This new PylRS has been demonstrated to effectively incorporate a variety of NCAAs,^313,314^ though the simple transfer of specificity for certain NCAAs from mb/mmPylRS variants to maPylRS is not always feasible.^315,316^ Furthermore, the tRNAs of *Methanosarcina* PylRS and *ma*PylRS cannot be freely interchanged. While *mm*PylRS can charge *ma*PylT, no tRNA aminoacylation was observed *vice versa*. This discovery has opened new avenues for the creation of mutually orthogonal PylRS pairs. Such orthogonality has been successfully achieved by introducing modifications in the variable loop of *ma*PylT, resulting in mutually orthogonal PylRS pairs in *E. coli*,^312^ mammalian cells,^317^ and yeast.^318^

When used in conjunction with other aaRS, such as *mj*TyrRS, employing several mutually orthogonal pairs theoretically supports the incorporation of multiple distinct NCAAs. Indeed, exploration of various uncharacterized PylRS has led to the construction of quintuple mutually orthogonal pairs, although the incorporation of NCAAs into proteins with those quintuple orthogonal pairs has not been demonstrated.^319^ Notably, the competition of suppressor tRNAs with the release factor usually leads to a truncated translation product alongside the desired readthrough product. This reduces the efficiency when multiple UAG (amber) codons or even multiple different stop codons, within a single mRNA transcript drastically.^320^ In *E. coli*, release factor 1 mediates termination at the UAG and UAA stop codon (ochre), whereas RF2 acts on UGA (opal) and UAA codons as well. Thus, disrupting RF1-stop codon interaction can greatly increase UAG suppression efficiency with only minor effects on overall translation termination. The overexpression of the C-terminal domain of ribosomal protein L11 as a competitor of RF for the ribosome increased the efficiency of the incorporation of N^ε^-acetyl-l-lysine at three sites.^321^ In a more drastic approach RF1 was completely knocked out, and rendering the organism incapable of terminating at UAG codons, rendering the organism incapable of terminating at amber codons.^320^ Additionally, recoding of essential amber codons or the entire genome from amber to ochre codons together with an RF1 knockout, resulted in *E. coli* strains such as 321.ΔA allowing for higher NCAA incorporation efficiencies.^322^ Since then, the strain 321.ΔA has been a scaffold for several further optimizations and applications.^323−325^

In eukaryotes, the situation is more intricate as all stop codons share the same release factor. Nevertheless, engineering approaches have yielded modified eRF1 with a single-point mutation that reduces its affinity for the amber stop codon, enhancing the suppression efficiency of the amber stop codon when coupled with an optimized PylRS/PylT pair by up to 20-fold.^326^ A similar strategy for modifying eRF1 has yielded promising outcomes for the suppression of various stop codons within mammalian cells notably increasing the efficiency from 0.78% to 11.6% through the implementation of triply orthogonal pairs.^327^

Due to their open and versatile nature, most technologies for incorporating NCAAs can be seamlessly applied in CFPS systems by simply supplementing the CFPS with the requisite components. Early on, the ability to effortlessly introduce precharged tRNA into the translation reaction in lysates from *E. coli* or rabbit reticulocytes played a pivotal role in shaping the development of cotranslational NCAA incorporation. Unlike living cells, CFPS is not bound by constraints related to cell viability or cellular membranes, making it particularly remarkable for its ease of manipulation when it comes to controlling reaction conditions. For example, it allows for relatively straightforward, residue-specific NCAA incorporation by utilizing amino acid-depleted lysates and supplementing them with an amino acid mixture containing the desired NCAAs.^328^

The zenith of user-defined CFPS is the PURE system, a cell-free system reconstructed from highly purified molecular components, including ribosomes, translation factors, and RNA.^329,330^ By supplying translation components such as tRNAs individually, PURE enables users to directly modify the genetic code, allowing for facile sense codon reassignment.^331−333^ Using a fully synthetic tRNA pool of 32 tRNAs, it has been possible to incorporate three different NCAAs.^334^ However, it is important to note that synthetic tRNA leads to lower protein yields in PURE compared to using native tRNA pools.^332^ While the defined nature of PURE makes it a valuable tool for unraveling the molecular functions of the translation machinery, it tends to be costlier and yields proteins at a moderate rate. More commonly, CFPS focuses on using cell lysates. In this scenario, sense codon reassignment can be achieved by selectively sequestering specific tRNAs from the lysates, as demonstrated for both *E. coli* lysate and the eukaryotic *L. tarentolae* lysate.^335^

Nevertheless, the most prevalent approach to NCAA incorporation involves stopping codon suppression. Besides the possibility to externally supply suppressor tRNAs, they can also be coexpressed. Therefore, ribozymes that self-cleave into functional tRNA are transcribed.^336^ Due to the resilience of CFPS to otherwise harmful substances and conditions, toxic amino acids such as canavanine can be incorporated.^337^ Also, the use of aaRS concentration far above physiological concentrations can be applied, as shown for a PylRS from Archeon ISO4-G1, that allowed the otherwise inefficient incorporation of N^ε^-(p-ethynylbenzyloxycarbonyl)-l-lysine, yielding over 1 mg/mL protein.^338^

Just as in cells, the competition between the release factor and suppressor tRNA can lead to truncated protein products. Various strategies have been devised to address this issue. One example is the utilization of an RF1-specific RNA aptamer to deactivate RF1 in the PURE reaction. Another approach involves using recoded organisms for lysate production, as exemplified by the 321.ΔA strain mentioned earlier. After further refinement, 321.ΔA based CFPS enabled the incorporation of an NCAA at up to 40 positions with yields of nearly 100 μg/mL in *E. coli* CFPS.^192,339^

In the realm of eukaryotes, cell-line engineering has been employed to generate stably transfected CHO cells for lysate preparation, which readily include the TyrRS or PylRS.^203^ While CFPS predominantly adapts approaches originally developed for living cells, it also possesses the potential to drive the development of novel technologies that can be applied in cell-based expression systems. For instance, the coexpression of suppressor tRNA with an sfGFP reporter has allowed for the rapid characterization of new suppressor tRNAs.^340^ Additionally, through the compartmentalization of cell-free reactions within liposomes and the application of fluorescence-activated cell sorting (FACS), the *in vitro* evolution of aaRS has become feasible. This approach has been exemplified with a PylRS exhibiting enhanced efficiency for the incorporation of N-benzyloxycarbonyl-l-lysine both *in vivo* and *in vitro*.^341^

## 5 Combining CFPS, Nonconical Amino Acid Conjugation, and Cell-Free Metabolic Engineering

We have framed past results to highlight the future importance of unnatural amino acids and cell-free synthesis to move beyond the boundaries of nature to produce high-quality drugs and address precision manufacturing, especially the need to combine these methods.

Site-specific coupling methods of payloads are easily integrated during cell-free protein synthesis. The components necessary for NCAA incorporation can be directly added to the translation machinery. In contrast to cell-based expression, the NCAA does not need to cross any cell membranes. In this regard, Zimmerman et al. established a cell-free protein expression system based on *E. coli* for production of ADCs by using the amber stop codon (UAG) suppression to integrate the noncanonical amino acid para-azidomethyl-l-phenylalanine (pAMF) at a chosen position.^342^ The introduction of the NCAA with subsequent coupling to a chosen drug eliminates heterogeneity and instability that might occur by using stochastic conjugation via endogenous lysine and cysteine residues. By using noncanonical amino acids, the actual antibody does not need to be modified, e.g., by disulfide shuffling or additional cysteines. Also, the position of the amber stop codon in the gene sequence can be located elsewhere. There is no limitation using only N- or C-terminal tags.

The position of the conjugation site influences ADC properties such as the stability, conjugation efficiency, antigen-binding, and internalization. The ability to freely choose the position of the conjugation benefits the mentioned parameter. Therefore, a dual fluorescence reporter system for the straightforward assessment of amber suppression and connected functionality is useful. Such an assay was developed by Krebs and Rakotoarinoro et al. to determine the influence of the position of the amber stop codon to the activity of a scFv.^343^ Similar approaches might also work for other protein classes.

In the beginning of the development of cell-free synthesized ADCs, the integration efficiency of the NCAA was a limiting factor to the total amount of full-length ADC. Nowadays, with the vast amount of different orthogonal systems and the NCAA this limitation is circumvented. Still, a suitable orthogonal system has to be identified. Another limiting factor is the DAR of cell-free synthesized ADCs. Considering only one amber stop codon in each heavy chain, resulting in one conjugation site, the maximum DAR is two. Zimmermann et al. found in their study DAR values between 1.2 and 1.9, confirming the low DAR.^342^

A solution was presented in 2017. Yin et al. engineered an RF1 mutant *E. coli* strain in which RF1 is sensitive to OmpT protease cleavage.^344^ This approach allowed normal cell growth for the highly active extract. Furthermore, this idea was much simpler compared with knocking out RF1 completely and replacing hundreds of TAG stop codons with TAA, allowing RF2 to replace RF1. Using their modified cell extract, Yin et al. expressed trastuzumab with multiple NCAAs integrated and coupled to DBCO-PEG4-maytansine. Depending on the number of stop codons, they detected DARs of 1.77 for one stop codon, 3.83 for two stop codons, 5.82 for 3 stop codons, and 7.43 for four stop codons. The expression of the construct harboring four amber stop codons showed a decrease in the efficiency. Reasons might include suppression efficiency and general stability of the ADC. They further evaluated the influence of higher DARs on potency using a panel of different HER2 expressing cell lines. Interestingly, they observed cell line dependent effects. On the one hand, the increasing DAR had no influence on the cell line SKBR3, whereas the cell line MDA-MB-453 was only effectively killed with ADCs that had a DAR of four or higher. In general, a higher DAR resulted in a higher potency of the ADC. HER2-negative cells were not killed independently of the DAR.

The advantages of incorporating multiple conjugation sites into the heavy chain was further utilized to create a hybrid *in vivo*/*in vitro* system^345^ with correctly assembled antibodies with high titers. The IgG light-chain (LC) was expressed in a conventional recombinant *E. coli* expression system, engineered to have an oxidizing cytoplasm for disulfide bridging. The LC was afterward added to a cell-free reaction synthesizing the heavy chain (HC) with multiple conjugation sites. With this strategy, the advantages of both systems were combined: high titers and simple manufacturing and incorporation of multiple NCAAs in a correctly assembled IgG.

The cell-free environment does not only provide a scaffold for protein synthesis but also for the synthesis of smaller molecules, since cell-free metabolic engineering has the potential to overcome some limitations of existing cell-based systems.^346^

In its most basic form, the homogeneous enzymes present in the cell-free extracts can be used to perform biotransformation reactions as shown for the generation of the antibiotic cefminox^347^ in Streptomyces extracts. Due to the open nature of CFPS platforms, the elucidation of biosynthetic routes can be achieved by adding labeled substrates or specifically downregulating certain pathways by the addition of inhibitors as shown for the preservative ε-poly-l-lysine.^348^ However, the true potential of cell-free extracts is shown in the development and improvement of new biosynthetic pathways in cell-free metabolic engineering (CFME). Here whole synthesis pathways can be engineered and composed from modules or enzymes that are (i) already present in the native cell-extract, (ii) heterologously expressed before lysate preparation, or (iii) synthesized in situ through CFPS.^349^ Additionally, the reaction is always accessible for the supplementation of further reagents to adjust the reaction conditions. In contrast to cell-free biosynthesis based on purified enzymes, the cumbersome preparation of pathways in which enzymes can be eliminated. Furthermore, the endogenous components in the cell extract can be used for cofactor regeneration allowing more efficient use of the starting material.^350,351^

Compared to traditional cell-based metabolic engineering approaches, CFME has much shorter DBTL cycle times that allow for quick elucidation and fine-tuning of the modules of synthetic pathway. These possibilities were impressively shown by Karim et al. (2016)^352^ through the construction of 17-step pathway for n-butanol synthesis in an *E. coli* cell-free extract. In a combinatorial approach, several enzymes were pre-expressed before lysate preparation, and the lysates containing these enzymes were mixed. The synthetic pathway further made use of enzymes and cofactors natively present in the *E. coli* extract including additionally *in situ* synthesized enzymes.^352^ Apart from other prominent examples like DHAP,^353^*E. coli* extract and *in situ* synthesized enzymes,^352^ and 2,3-butanediol,^354^ more complex molecules have not been investigated. Combining polyketide synthase modules, enzymes for substrate generation, and cofactor regeneration in an *E. coli* cell-free environment, the synthesis of triketides was facilitated. These molecules can be used as building blocks e.g., for anticancer drugs.^355^

Though most of these approaches utilize prokaryotic (mostly *E. coli*) extracts, CFME is not limited to those. Recent studies show that lysates from *S. cerevisiae* or tobacco cells are well-suited for metabolic engineering and the subsequent synthesis of metabolites.^172,356^

When it comes to the preparation of NCAAs, the exploration of biosynthetic routes for the NCAA synthesis holds considerable promise. While, to our knowledge, no cell-free approaches for this purpose currently exist, there are examples in classical expression systems. Establishing a biosynthetic pathway for NCAAs could be more sustainable than traditional chemical synthesis, and it holds the potential to reduce cultivation costs by eliminating the need to supplement the culture medium with high concentrations of NCAAs. As such, the exploration of biosynthetic routes for NCAA synthesis holds promising prospects. A pioneering example of such biosynthetic pathways were demonstrated by the creation of a fully autonomous *E. coli* strain with para-amino-phenylalanine as the 21st amino acid, achieved by introducing three genes from *Streptomyces venezuelae*.^357^ Other instances of NCAAs produced via engineered biosynthetic pathways include 5-hydroxyproline (5-HTP),^358^ DOPA,^358−360^ and S-allyl-homocysteine, the latter two being particularly noteworthy for bioconjugation applications.^361^

## 6 Discussion and Future Outlook

Throughout history humanity has been screening nature for therapeutic agents and has come a long way from the early trial and error, via isolation of active compounds and proteins, to careful biomanufacturing of highly specific, or even personalized, drugs. Biopharmaceuticals, including pharmaceutical proteins, are here to stay. Currently there are more than 7 800 biopharmaceutical products in clinical development globally, of which over 1000 have reached phase III clinical trials.^16^ The large pipeline, and additionally regulatory experience accumulated in the past few years, also due to the COVID-19 pandemic, should accelerate the speed of drug development and approval processes for future medicines. In addition, due to expiring patents, additional biosimilar molecules will enter the market at the same time. As such, production platforms, including bioprocessing and DSP, need to be ready for this increased need.

The current successes in the clinic of conjugated peptides and proteins can be ascribed to the successful alliance of biology and synthetic chemistry. One major advance has been to expand beyond the cell and perform both biomanufacturing in cell-free systems and to expand the genetic lexicon.

With the availability of high-quality data, the relationship of biological data sets, machine learning algorithms, and the utilization of language models to train artificial intelligence is under rapid developments for current applications in metabolic engineering and protein design.^362−368^ However, in order to advance modern medicine and unlock the potential even further for biomanufactured therapeutic agents, an additional merger of automation engineering, computational chemistry, quantum computing,^369^ and additional artificial intelligence tools is needed.

Such a merger would result in the need to design novel *in silico* extrapolative tools and enhanced high-throughput methods. We could argue that such initial approaches have shown great promise for nonconjugated antibody drugs; however, we are still lacking sufficient tools for bioconjugated drugs. The current state-of-the art in ‘cell-free protein synthesis’ and ‘cell-free metabolic engineering small molecule synthesis’ presented here indicates a future merger of the two approaches in order to enable screening the vast amount of all possible combinations of the various modular components to emerge from the advancements in structure–activity studies in the future. Further advances in predictive tools for *in vivo* drug delivery, efficacy, novel drug target discovery, and metabolic clearing models are needed to shave down the enormous landscape of possible drug molecules. In parallel, drug developability has to be improved and re-evaluating risk management through postmarketing surveillance is needed. Despite these hurdles, the role of cell-free systems and NCAAs in shaping the future of drug design, screening, and manufacturing has only just begun.

## Abbreviations

aaRS: Aminoacyl-tRNA synthetase
ADC: Antibody–drug conjugate
BHK: Baby hamster kidney (cells)
CFPS: Cell-free protein synthesis
CFME: cell-free metabolic engineering
CHO: Chinese hamster ovary (cells)
CNTF: Ciliary neurotrophic factor
CRISPR: Clustered regularly interspaced short palindromic repeats
DAR: drug antibody ratios
DBTL: Design, build, test, and learn
DNA: Deoxyribonucleic acid
DOPA: l-3,4-Dihydroxyphenylalanine
DSP: Downstream processing
*E. coli*: *Escherichia coli*
EGF: Epidermal growth factor
EGFR: Epidermal growth factor receptor
EMA: European Medicine Agency
EPO: Erythropoietin
FDA: Food and Drug Administration
GFP: Green fluorescent protein
GlcNAc: N-Acetylglucosamine
GM-CSF: Granulocyte-macrophage colony-stimulating factor
GRAS: Generally regarded as safe
HC: Heavy chain (of an antibody)
*H. polymorpha*: *Hansenula polymorpha*
IFN: Interferon
IRES: Internal ribosome entry site
IVT: *In vitro* transcription
IVTT: *In vitro* transcription and translation
*K. pastoris*: *Komagetaella pastoris*
*L. tarentolae*: *Leishmania tarentolae*
LC: Light chain (of an antibody)
LeuRS: Leucyl-tRNA synthetase
mAB: Monoclonal antibody
mRNA: mRNA
MS: Mass spectroscopy
MTGse: Microbial transglutaminase
MW: Molecular weight
NCAA: Nonconical amino acid
NHS: N-Hydroxysuccinimides esters
NMR: Nuclear magnetic resonance
*P. pastoris*: *Pichia pastoris*
PAMF: Para-azidomethyl-l-phenylalanine
PAS: As in PASylation: polypeptide chain of Proline, Alanine, and Serine
PCR: Polymerase chain reaction
PEG: Poly(ethylene glycol)
PK/PD: Pharmacokinetics and pharmacodynamics
PMSF: Phenylmethylsulfonyl fluoride
POX: Poly(2-oxazoline)
PTM: Post translational modification
PURE: Protein synthesis using purified recombinant elements
PylRS: Pyrrolysine-tRNA synthetase
RBD: Receptor-binding domain
RF: Release factor
RNA: Ribonucleic acid
RT: Reverse transcriptase
*S. cerevisiae*: *Saccharomyces cerevisiae*
*S. gregaria*: *Shistocera gregaria* (desert locus)
SARS: Severe acute respiratory syndrome
scFv: Single-chain fragment variable [of an antibody]
SDS-PAGE: Sodium dodecyl sulfate–polyacrylamide gel electrophoresis
TEA: Techno-economic assessment
tRNA: tRNA
TrpRS: Tryptophanyl-tRNA synthetase
TyrRS: Tyrosyl-tRNA synthetase
UPC: Unit production cost (total annual operation cost/annual mAb produced)
